# Systematic surveillance tools to reduce rodent pests in disadvantaged urban areas can empower communities and improve public health

**DOI:** 10.1038/s41598-024-55203-5

**Published:** 2024-02-24

**Authors:** Adedayo Michael Awoniyi, Ana Maria Barreto, Hernan Dario Argibay, Juliet Oliveira Santana, Fabiana Almerinda G. Palma, Ana Riviere-Cinnamond, Gauthier Dobigny, Eric Bertherat, Luther Ferguson, Steven Belmain, Federico Costa

**Affiliations:** 1 Instituto de Saúde Coletiva, Universidade Federal da Bahia, Salvador, BA 40110-040 Brazil; 2 Instituto de Biologia, Universidade Federal da Bahia, Salvador, BA 40170-115 Brazil; 3 Centro de Pesquisas Gonçalo Moniz, Fundação Oswaldo Cruz, Salvador, BA Brazil; 4 Data Management, Analytics and Products (DMAP), Health Information and Risk Assessment Unit (HIM), PAHO Health Emergencies, Washington, DC USA; 5 French Institute of Research for Sustainable Development (IRD), UMR CBGP, Montpellier, France; 6 Pasteur Institute of Madagascar, Plague Unit, Antananarivo, Madagascar; 7 Department of Pandemic and Epidemic Diseases, World Health Organization WHO, Geneva, Switzerland; 8 Department of Environmental Health Services (DEHS), Ministry of Environment and Natural Resources, Nassau City, Bahamas; 9 Natural Resources Institute, University of Greenwich, Chatham Maritime, Kent, ME4 4TB UK; 10 Department of Epidemiology of Microbial Diseases, Yale School of Public Health, New Haven, CT06511 USA; 11 Lancaster Medical School, Lancaster University, Lancaster, LA1 4YW UK

**Keywords:** Community engagement, Sustainable rodent management, The Bahamas, Urban rodent survey, Waste management, Zoonoses, Animal migration, Behavioural ecology, Community ecology, Ecological epidemiology, Population dynamics, Urban ecology

## Abstract

Rodents are notorious pests, known for transmitting major public health diseases and causing agricultural and economic losses. The lack of site-specific and national standardised rodent surveillance in several disadvantaged communities has rendered interventions targeted towards rodent control as often ineffective. Here, by using the example from a pilot case-study in the Bahamas, we present a unique experience wherein, through multidisciplinary and community engagement, we simultaneously developed a standardised national surveillance protocol, and performed two parallel but integrated activities: (1) eight days of theoretical and practical training of selected participants; and (2) a three-month post-training pilot rodent surveillance in the urban community of Over-the-Hill, Nassau, The Bahamas. To account for social and environmental conditions influencing rodent proliferation in the Bahamas, we engaged selected influential community members through a semi-structured interview and gathered additional site-specific information using a modified Centers for Diseases Control and Prevention (CDC) exterior and interior rodent evaluation form, along with other validated instruments such as tracking plates and snap trapping, to test and establish a standardised site-specific rodent surveillance protocol tailored for the Bahamas. Our engagement with community members highlighted poor disposal of animal and human food, irregular garbage collection, unapproved refuse storage, lack of accessible dumpsters, poor bulk waste management, ownership problems and structural deficiencies as major factors fuelling rodent proliferation in the study areas. Accordingly, results from our pilot survey using active rodent signs (that is, the presence of rodent runs, burrows, faecal material or gnawed material) as a proxy of rodent infestation in a generalized linear model confirmed that the variables earlier identified during the community engagement program as significantly correlated with rodent activities (and capturing) across the study areas. The successful implementation of the novel site-specific protocol by trained participants, along with the correlation of their findings with those recorded during the community engagement program, underscores its suitability and applicability in disadvantaged urban settings. This experience should serve as a reference for promoting a standardised protocol for monitoring rodent activities in many disadvantaged urban settings of the Global South, while also fostering a holistic understanding of rodent proliferation. Through this pilot case-study, we advocate for the feasibility of developing sustainable rodent control interventions that are acceptable to both local communities and public authorities, particularly through the involvement of a multidisciplinary team of professionals and community members.

## Introduction

Rodent proliferation is a major threat to human communities, associated with disease transmission, disruption of the ecosystem including extinction of native species, destruction of infrastructures and household goods, as well as significant agricultural loss^1–5^. Relationships between poor urban socio-environmental conditions with rodent pests and human health are widely recognised, especially in Low and Middle-Income Countries (LMICs)^6,7^. In these countries, basic knowledge about rodent population dynamics, migration patterns and behaviour is often lacking^8^. For example, disadvantaged communities (e.g. slums, informal settlements and shantytowns) are essentially characterised by poverty, unrecognised land-use policy, precarious infrastructure, poor sanitation systems and generalised insalubrities. These conditions support rodents’ easy access to harbourage, food and water sources, hence providing them with lodge and boarding, while encouraging their proliferation and movement between different habitats and subsequently resulting in potential transmission of rodent-borne diseases to humans^9,10^. Specifically, residents of these environments are exposed to probable pathogen spillover from rodents^11^ leading to outbreaks of rodent-borne diseases such as plague as witnessed in Madagascar^12^, Lassa fever in Nigeria^13^, increasing numbers of hantavirus cases in the Americas^14,15^ and leptospirosis, a disease estimated to affect almost one million people worldwide^16^, with most cases mainly described from Latin America and Asia^17^, with an under-documented burden in Africa^18^. They may also serve as known reservoirs for other important vector-transmitted and environmental pathogens, such as *Toxoplasma*, *Schistosoma, Rickettsia*, *Salmonella*, *Bartonella, Borrelia* etc.^19^. Rodent pests have also been linked to mental health problems in many urban communities^20,21^, which may be related to disturbed sleep, rodent bites and psychological trauma^22–24^.

A wide range of control intervention strategies have been directed towards the management of rodents. Unfortunately, most of those interventions have not yielded long-term solutions^25,26^, partly due to limited resources and a lack of robust surveillance systems^27,28^ (that is, the procedures used for detecting the presence and or quantity of rodent infestation and target species, and the factors influencing their population in the environment in an attempt to propose a sustainable rodent management measures). Over-dependence on non-locally adapted control methods essentially based on chemicals have proven to sometimes produce unwanted and adverse side-effects^29^, and become less effective over time^30,31^ probably because they lack the involvement of local residents during intervention efforts^32–34^. As evidenced by Colombe et al.^19^, there also exists a shortage of rodent experts as well as active rodent surveillance and management programs in most countries. In addition, where such programs exist, they often fail to monitor the impact of management actions on rodent proliferation in the long term and assess their cost-benefits balance^35^. In some instances where active national programs exist, surveillance is often conducted too late, in a reactive manner, thus failing to prevent outbreaks and/or high impact damage^36^. Furthermore, they usually consist in “top-down” methods, i.e. hierarchical approach with centralised decision-making power, that neglect the inclusion of other stakeholders/parties and finally fail^37^. Also, these methods lack standardisation of proxies (such as rodent runs, burrows, faecal or gnawed material and rodent capturing) and results across study areas—specifically using reports of rodent sightings to evaluate infestation”, and ignoring the other important actors to develop innovative control strategies^28,30^.

A typical example of a national rodent management program with the common shortcomings highlighted above is found in the Bahamas. Here, conventional practice relies on reports of rodent sightings by residents and a few experts for evaluating the level of rodent infestation, followed by systematic use of anticoagulant poisons with little monitoring of rodent activity before and after interventions^30^. At the invitation of the Bahamas, the World Health Organization (WHO) and the Pan American Health Organization (PAHO) aimed to work with local authorities to implement an urban rodent surveillance training workshop, using the country as a pilot study site to develop an effective and sustainable rodent surveillance strategy that could be exported in other countries. This paper describes and discusses this experience which aimed at (1) understanding specific socio-environmental drivers of rodent proliferation in the Bahamas; (2) developing and testing tools for rodent surveillance actions; and then (3) suggesting site-specific management protocol against rodent populations proliferation based on pilot-surveillance outcomes. We thus present an innovative framework which incorporates active community participation and multidisciplinary team engagement to develop strategic guidelines for a standardised surveillance protocol, perform rodent surveillance training and conduct a post-training rodent surveillance to achieve long-term rodent population management. The outcomes of this pilot study provide a sustainable and socio-culturally appropriate intervention framework that could be adapted to other similar contexts found across poor urban areas in other LMICs where rodent pests are having significant negative impacts on human livelihoods, public health, both physical and mental wellbeing, as well as food and nutrition security, environmental sanitation and economic productivity.

## Results

### Rodent training workshop and community meeting

We leveraged the 8-week multidisciplinary pre-training meetings to evaluate the techniques used for conducting rodent surveillance in poor urban communities of Nassau, the Bahamas. It was noted that rodent infestation detection in these communities has been performed using “top-down” methods from the 1960s. Precisely, relying on spontaneous reports of rodent sightings by the residents and vector control experts during visits conducted in the day (afternoon to evening) and over a limited duration (≤ 4 h per day)^30^. From these identified shortfalls, we developed a site-specific standardised rodent surveillance protocol (Supplementary Annex I—“Guideline for the Integrated Rodent Management (IRM) in the Bahamas” and Supplementary Annex II—“A modified CDC exterior and interior surveillance form”) that appeared particularly adapted to poor urban settings and offered improvement over the previous procedure by factoring the period that rodents are most active (night/early morning) into surveillance activity. We trained (for 8 days) twenty-three (23) local participants (mostly from the DEHS, Ministry of Environment and Natural Resources, which is responsible for vector control in the Bahamas) to implement this protocol in an autonomous manner.

**Figure 1:**
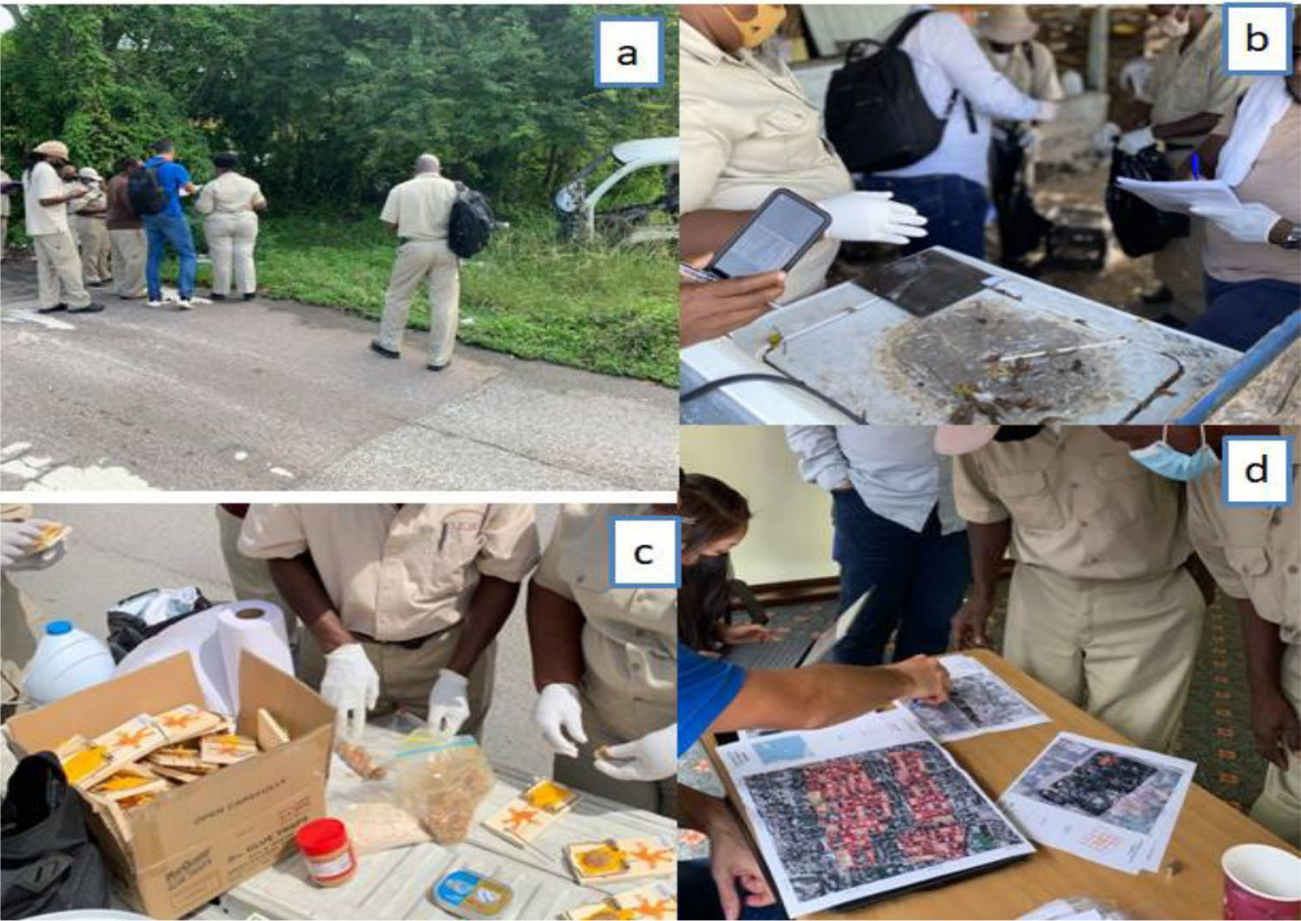
Cross-section of some of the participants and training facilitators during the WHO/PAHO Bahamas rodent surveillance training workshop (**a**) team briefing during the exterior rodent surveillance exercise, (**b**) the team photographing and examining the TP for a possible rodent mark, (**c**) the participants preparing and apportioning appropriate baits to the STs before the STs are setup for rodent capturing, and (**d**) training facilitators explaining how to go about collaborative mapping to some of the workshop participants. Photographs by T. Dorji.

Systematically, we thoroughly explained each component of the surveillance protocol to the participants during both theoretical and practical rodent surveillance training sessions. Briefly, we modified the CDC exterior and interior rodent evaluation form to suit our study area. In addition, participants received hands-on practical training on the use of tracking plates and snap trapping (Fig. 1a–c). Also, they were trained on how to store data in REDCap. We created an account for the project (the pilot surveillance) as well as a login for each participant, which they could use to report results of their own environmental survey during and after the training period.

**Figure 2:**
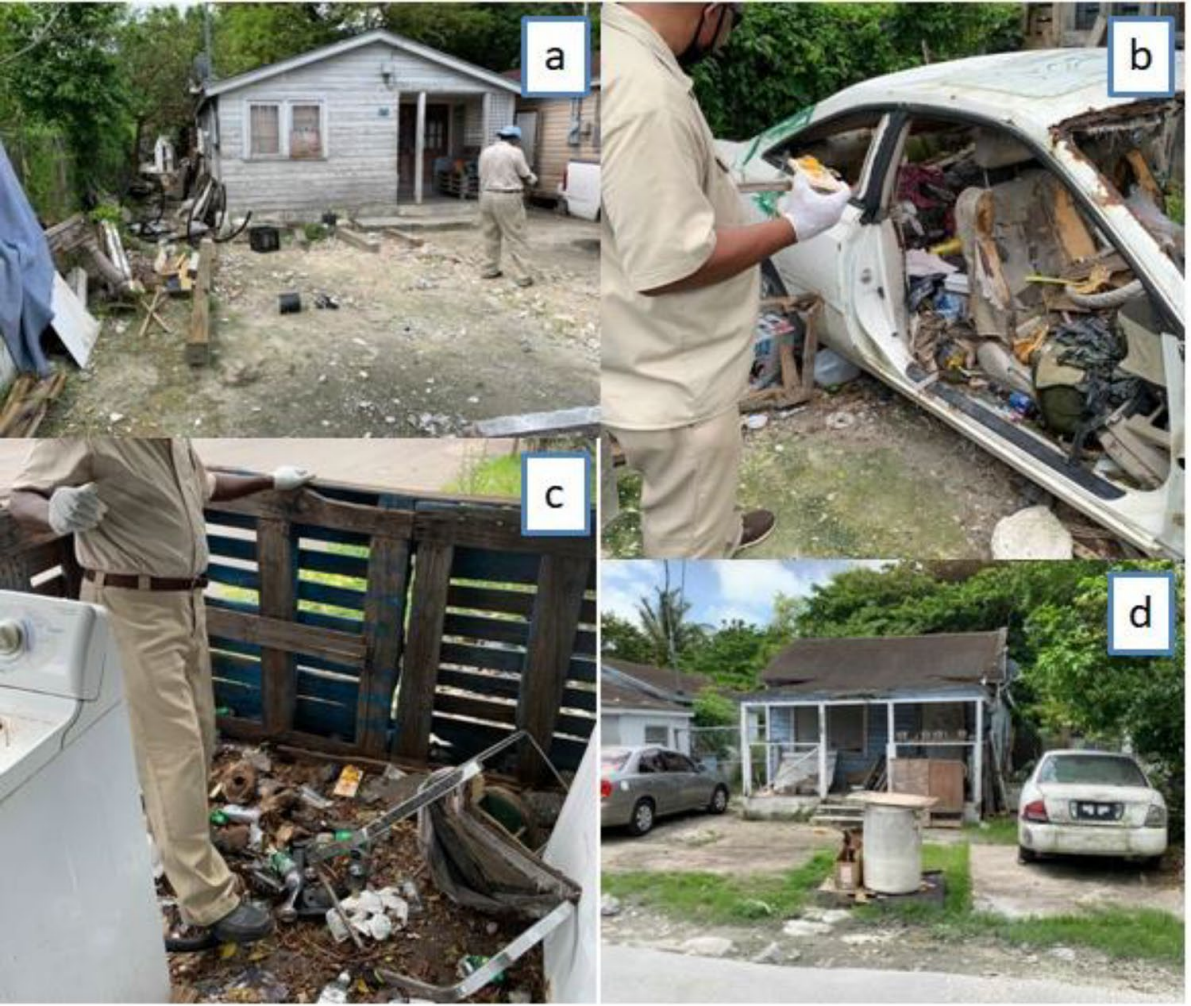
Some of the conditions that may influence rodent population as observed here in the Bahamas during the WHO/PAHO Bahamas rodent surveillance training workshop: (**a**) residence with construction materials and other debris, (**b**) residence with a wrecked/abandoned vehicle, (**c**) residence with an unkempt surrounding, and (**d**) residence bordering abandoned building and vehicle. Photographs by T. Dorji.

As part of the training exercise, the DEHS representatives, WHO and PAHO officials and the training facilitators (experts) met with representatives of the local community (11 focal persons selected from the surveyed households for comprehensive community engagement). The purpose of this initial meeting was to extensively—(1) evaluate the community perception about rodent infestation using a collaborative mapping approach, where the local community members were tasked to identify areas with rodent-related problems, and (2) discuss the problems responsible for the rodent infestation and recommend possible solutions to the identified problems jointly with the community members, the training facilitators and the DEHS, WHO and PAHO officials (Fig. 1d). Summarily, from the community meeting report, the majority of the participants (at least 8 out of the 11 participants) indicated that certain areas were well-conserved with reasonably satisfactory waste disposal practices, while others displayed characteristics such as: ownership problems (houses with unknown owners, often under lock and as a result preventing accessibility during rodent surveillance/control); haphazard waste disposal and irregular pick-up schedules leading to garbage pileups; lack of access to dumpsters; abandoned houses; non-collection of bulk waste e.g. derelict vehicles and construction materials offering rodents harbourage sources (Fig. 2a–d); and socio-cultural and economic differences (especially household income-related differences, with some households lacking funding to support appropriate self-waste disposal) among residents (Table 1). Ambiguity of waste management protocol between the residents and environmental health officers was also noted during the meeting (e.g. inhabitants claiming they lack sufficient dumpsters and require a higher frequency of garbage pickup vs. waste managers saying there were enough dumpsters and that pick-ups were carried out quite frequently). As a result, a majority of the participants suggested various intervention measures, including the provision of additional dumpsters in easily accessible locations, removal of bulk waste, and the implementation of community educational awareness initiatives in clear and diverse languages understood by the residents, among other recommendations (Table 1).

**Table 1:** Synopsis of the feedback gathered during the community collaborative meeting and mapping session.

Theme	Feedback from the majority of community members	Feedback from the minority of community members
Factors capable of influencing rodent proliferation	Disposal of food (leftover) in open spaces Irregular garbage collection Disposal of garbage in open bins Lack of accessible dumpsters Household ownership problems Non-collection of bulk waste Lack of funds for proper self-waste disposal	Poor enforcement of environmental laws Erosion of personal pride Overcrowding Language barriers among some residents Overreliance on government for cleanup exercises
Suggested solutions	Install dumpsters in strategic locations accessible to the residents Removal of bulk waste e.g. derelict vehicles by the DEHS Install signage stating “no dumping on vacant properties” Implement additional community initiatives focused on educating and empowering the community and children using diverse languages e.g. use media as a strategic tool to raise awareness about rodent control efforts in the community Owning cats	Enforce effective property management, mandating property owners and residents to ensure adequate cleanliness Establish partnership with Community-Based Organisations, Churches and Schools to organise cleanup campaigns and promote sanitation awareness within the community Tree planting
Collaborative mapping of rodent-related issues at the area level	Areas 1 & 6—ownership problem & haphazard waste disposal Area 2—decent area with a moderately satisfactory waste disposal Area 3—derelict vehicles & haphazard waste disposal Area 4—presence of garbage; derelict vehicles and abandoned properties (houses) Area 5—some garbage Area 7—informal community & abundance of undocumented car garages	

Feedback from the majority of community members reflects the opinions of at least 8 out of the 11 participants, while feedback from the minority represents the opinions of 3 participants or fewer respectively.

### Rodent surveillance points and assessment of the factors driving rodent proliferation

We simultaneously conducted pilot rodent surveillance using two independent methods (exterior and interior household rodent survey^38^ and rodent trapping^39^) that have been proven as proxies for evaluating household rodent infestation in 713 households/points across the study community, with each household representing a sampling point or unit.

We retrieved complete information about 457 points, with data on the remaining 256 points being incomplete, missing or inaccessible (e.g. the houses containing the points were closed). In total, we trapped 106 rodents (including 36 *Rattus norvegicus,* 27 *R. Rattus*, 13 *Mus musculus*, 10 *Rattus* spp.—suspected *R norvegicus*- and 20 unidentified individuals) from 457 trap nights, yielding 23.2% trapping success. Similarly, we conducted extensive three months long post-training surveillance campaign (June–August 2022) in the study areas and recorded an apparent rodent infestation across the seven study areas (areas 1–7), with contrasted degrees of infestation showing significantly higher rodent infestation in study areas 3, 4 and 7 (Table 2).

**Table 2:** Summary of the generalized linear model showing the variables that were associated with rodent proliferation in the Bahamas.

Predictors	Signs of household rodent infestation		
	Odds ratios	CI	P
(Intercept)	0.09	0.04–0.19	** < 0.001**
Area [2]	0.55	0.20–1.50	0.192
Area [3]	0.36	0.13–1.00	**0.047**
Area [4]	0.27	0.08–0.84	**0.021**
Area [5]	0.71	0.34–2.91	0.532
Area [6]	0.99	0.31–2.64	0.855
Area [7]	0.07	0.02–0.28	**< 0.001**
Residence with unapproved refuse storage [Yes]	2.29	1.22–4.27	**0.008**
Residence with exposed garbage [Yes]	2.31	1.20–4.45	**0.016**
Residence with source of animal food [Yes]	2.30	1.11–4.17	**0.021**
Residence with other sources of food [Yes]	1.76	0.89–3.53	0.096
Residence with bulk wastes [Yes]	2.06	1.00–4.21	**0.045**
Residence with construction materials [Yes]	2.06	1.04–4.07	**0.047**
Residence with structural deficiencies [Yes]	3.09	1.52–6.25	**0.001**
Observations	457		
R^2^ Tjur	0.358		

Significant values are in bold.

To investigate factors possibly influencing rodent infestation using active rodent signs (i.e. rodent runs, burrows, faecal or gnawed material) as a proxy in the poor urban community under study, 16 variables with p-values of ≤ 0.15 from an initial separate analysis were included in the final model (Supplementary Annex III). Of these 16 variables, the final model (Table 2) retained eight (namely: area; residence with unapproved refuse storage; exposed garbage; source of animal food; other sources of food; bulk wastes; construction materials; and residence with structural deficiencies) with an Akaike Information Criterion (AIC) of 345.9, and the model with the fewest number of variables.

This final model (Table 2) showed that residence with unapproved refuse storage, exposed garbage, sources of animal food, bulk wastes, construction materials and structural deficiencies were significantly associated with rodent presence, with households with structural deficiencies (OR: 3.09 [1.52–6.25], p = 0.001) and exposed garbage (OR: 2.31 [1.20–4.45], p = 0.016) showing the highest association with rodent infestation level.

## Discussion

The co-implementation of the locally-adapted CDC rodent surveillance protocol (Supplementary Annex I) and those relying on tracking plates and snap trapping ensures that shortfalls of the former deficient surveillance techniques are compensated for, while also improving the accuracy and reliability of rodent infestation data in the poor urban communities^10,40^. The results of rodent survey generated by the training participants (a 3 months long pilot rodent surveillance) indicate that the current protocol is suitable and applicable in disadvantaged urban environments, in addition to encouraging local community participation in potential studies that could guide decision-making during rodent intervention programs. Central to the present paper, it also indicates that the participants truly understood and appropriated the concepts and methods proposed during the initial training session and were subsequently able to apply them appropriately in an autonomous manner as part of a rodent surveillance routine.

In the particular case of the Bahamas, the rodent-related problems (as identified by frequent residents’ reports of rodent sightings, gnawed materials, rodent holes and other active rodent signs observed during the pilot case-study) could be due to: (a) ownership issues, such as vulnerable areas lacking identifiable owners, which could hinder access during evaluation and interventions; (b) garbage pileup due to irregular waste pickup schedules and/or access to dumpsters; (c) ambiguity of waste management protocol between the residents and environmental health officers; (d) availability of harbourage sources facilitating rodent nesting and movements, such as construction materials, abandoned houses and derelict vehicles within the urban setting (Fig. 2a–d); and (e) cross-sociocultural (such as the differential or combined effects of Caribbean, American, Europe, Hispanic and African culture, religious beliefs, income levels, proximity to tourism centres, and ethnic values) differences among the local residents, with most residents lacking the capability to reside in an area with satisfactory social amenities and others lacking funding to support appropriate household-level sanitation that could reduce rodent infestation. All these factors were also clearly identified during the community group discussion, thus aligning with the results of our surveillance efforts. Also, the rodent infestation reported in Over-the-Hill could probably be due to rodents being able to take advantage of major sources of nourishment and harbourage from the local environment, as these have been demonstrated to encourage rodent proliferation and critical to rodent survival in urban environments^10,41–43^. The risk factors reported in our final model (Table 2), such as unapproved refuse storage, households with structural deficiencies and the presence of bulk waste and construction materials, are not unexpected, considering they all provide alternative sources of food, water and harbourage for rodents^44^. For instance, the socio-environmental conditions reported here to be significantly correlated with rodent proliferation are similar to those previously reported by Costa et al.^45^ and Awoniyi et al.^46^ in Brazil favelas.

Accordingly, our results confirm particularly strong rodent infestation with a varying levels of infestation across the study areas (Table 2), which could be due to the somewhat varying sanitation management and socioeconomic status of residents in the study areas^30^ (Table 1). Also, the overall reported 23.2% trapping success translates to significant rodent infestation, especially in study areas 3, 4 and 7. This appears higher than the 11.2% reported by Awoniyi et al.^46^ in disadvantaged urban communities of Salvador-Bahia, Brazil, 14.8% reported by Dossou et al.^47^ in disadvantaged communities of Benin Republic and considerably higher than the 1.6% reported by Shafie et al.^48^ in Malaysia.

In the present pilot case-study, the high number (N = 20) of rodents that could not be identified at the species level points towards poor or low research efforts targeted at rodents, and highlights the need for research in this field, including baseline knowledge such as rodent diversity assessment and the development of Great Caribbean Islands rodents identification key that could be easily used by operators in the field. Achieving this will add to the pool of existing knowledge about rodent diversity albeit supporting their appropriate control since species-specific features may be important to take into account when setting up locally adapted rodent management^49^.

Considering the low reported number of leptospirosis cases (i.e. 2 confirmed cases in 2 years, as mentioned by the representative of the MoH Epidemiological Surveillance Unit during the training), it contrasts strongly with the abundance of rodents on the Bahamas islands, therefore, we suspect that most cases of leptospirosis, together with other rodent-borne diseases, might be misdiagnosed and largely underreported. This may be due to the lack of a functional reference laboratory and trained personnel, in addition to the residents and local medical staff being poorly aware of leptospirosis risks and symptoms. Unfortunately, these caveats are not surprising as they have also been reported in other African and South American countries (Allan et al.^18^, Dobigny et al.^50^, Schneider et al.^51^ and Costa et al.^52^) where leptospirosis is not yet perceived as a disease of public health concern. As such, Rodrigues^53^ identified the inability to perform (early) diagnosis as a major setback encouraging the vicious circle of deficient leptospirosis control in Brazil. This is also expected to be true for other rodent-related and frequently overlooked or neglected diseases in the Global South, such as murine typhus, Lassa virus or hantavirus-mediated fevers^19^. Accordingly, this experience shows the urgent need to reinforce laboratory capacity in the country and to establish the real burden of rodent-borne diseases, for instance using seroprevalence studies as a first reasonable attempt.

Key lessons from this pilot study which aimed at informing the government of the Bahamas to control rodent proliferation and reduce the risk of rodent-borne diseases, are: (a) providing more access to dumpsters and frequent garbage/bulk garbage (vehicles, etc.) pickup, especially in the high-risk areas, (b) disseminating adequate information (i.e., in simple terms and local languages) about rodent-human contacts-related risk factors and possible precautions (this could be done in schools and other gathering places, such as religious sites), (c) establishing a clear definition and dissemination of waste management protocols among community members and environmental health officers to clarify their respective roles, (d) promoting social responsibility among the residents to always take actions that will guard against rodent propagation in the community (i.e. prompt disposal of waste using an approved trash container and keeping pets food and water away from the reach of rodents), and (e) promoting sustainable and partnership engagement among community members, local and state governments, especially the arm responsible for vector management in the communities. Importantly, associated educational and communication plans will have to take socio-economic constraints and priorities of the inhabitants into account in order to be widely accepted and subsequently appropriated.

The present pilot study was primarily used to develop and test a standardised surveillance protocol that would engage multiple stakeholders, conduct rodent surveillance training and implement pilot rodent surveillance activities. The pilot surveillance took place over a single season and a single urban setting, which likely limits our ability to fully understand the suitability of our protocol for a range of contexts found across urban areas in LMICs. However, several of the components presented have been tested and validated in other contexts^1,40,46,54,55^, thus making us confident in its easy and promising application in a wide range of other socio-cultural urban contexts over the world. To successfully adapt this protocol elsewhere, engagement of the local residents and other multi-sectoral stakeholders is essential and requires extensive concertation to fully understand which local socio-cultural constraints could be blocking progress and which leverages could be instrumental. In addition, baseline information about rodent species-specific life traits (e.g., population structure and dynamics, mobility/dispersal abilities, habitat preference, diet, resistance to rodenticide molecules, etc.), climatic and environmental conditions, geographic and physical contexts are needed in order to tailor any sustainable and locally-adapted intervention against rodent pests^34^. A further priority should be to carry out baseline surveillance of rodent-borne diseases circulating in both the rodent and human population, as there is a known correlation between rodent pest circulation and rodent-borne diseases in many contexts^1^. This will also be important to ensure that the developed protocol can be implemented safely by environmental health officers without making them at risk. Therefore, we suggest that future attempts be conducted in other LMIC urban contexts to adapt and further test the core components of this protocol to ensure that pest rodent surveillance and management can be done on a regular basis as a safe, sustainable and effective practice.

Besides, while the authors acknowledge that the involvement of more local residents, especially the religious and civil society leaders, as well as other multi-sectoral stakeholders (e.g., representatives of Ministries of Agriculture and Environment; healthcare staff; waste managers; local experts in biological and social sciences and local policymakers) might have improved the whole process, we were somewhat restricted due to resource constraints and the government’s directive to limit all physical gathering during the present pilot study. However, we believe the individuals trained and engaged during this pilot study represent a step in the right direction as they are tasked with the responsibility of transferring the newly acquired knowledge and abilities to other community members (train the trainer), especially those interested or involved in rodent-related programs.

Interestingly, here, we describe a context-specific and standardised rodent surveillance protocol that improves upon the shortcomings observed in the current reactive rodent surveillance protocol used in the Bahamas, and in many other LMICs. Currently used reactive protocols, such as rodent sightings, are prone to suggesting false negative or positive rodent infestation as some residents may exaggerate their observations while others may tolerate minor rodent infestation^56^, as well as greatly limiting standardisation of results, hence reliable comparison between monitored sites. In particular, inaccuracy is expected when applied protocols over-rely on reports of rodent sightings by the residents and/or vector control experts^30^ since such surveillance is usually conducted during the day and over a limited number of hours (≤ 4 h per day), thereby neglecting and/or overlooking rodent activities during the night/early morning, which are yet the most active period for rodents^57^ and should be taken into primary consideration when evaluating rodent infestation. Also, the current protocol offers an improvement over the application of the existing protocols (e.g. CDC’s) especially in many LMICs where buildings are not constructed in the standard block-like version. As a result, this protocol presents a flexible manner of satisfactorily conducting a more representative surveillance of most LMICs communities while also capturing specific variables that are peculiar to these environments.

Another probable limitation of this pilot study is our inability to collect pre- and post-data to facilitate a comprehensive comparison of our new protocol to the conventional “report of rodent sightings” method. However, we are of the opinion that comparing results from two different methods (pre-training: report of rodent sightings and post-training: results from the standardised rodent surveillance protocol) could affect the integrity of such comparison. Additionally, the data generated by the participants during and after the training (under minimal supervision) show that they understood and appropriated the standardised surveillance concept. It also shows that it is suitable for rodent surveillance in LMICs communities, as results from the pilot surveillance align with those suggested by the local residents during the community engagement meeting.

## Conclusion

This pilot study sought to explore the effectiveness of engaging multidisciplinary professionals and stakeholders to develop standardised rodent surveillance protocols. The outcomes showed that such an approach and the engagement of relevant stakeholders may be critical during rodent surveillance, and could be valuable to better guide future management interventions. Site-specific mitigation strategies are lacking in most urban disadvantaged communities, especially within LMICs where data on urban rodents and associated impacts are scarce. We argue that a better understanding of rodent proliferation in many locations across LMIC cities requires community engagement activities to ensure sustainable and culturally appropriate rodent control interventions that meet the needs of local communities and authorities.

Sadly, there is little doubt that ongoing urbanisation will amplify the ever increasing poor urban living conditions, thus facilitating the expansion of rodent populations in disadvantaged urban communities. This in turn may increase the risk of zoonotic disease spill-over and the emergence of new pandemics^9^. With multiple socio-economic problems, cost-beneficial rodent management solutions are urgently required to reduce such zoonosis risks whilst enabling improved livelihoods that can be more economically productive. As rodent research and expertise are often lacking or decreasing in many LMICs, we propose that investments in rodent-related research could significantly enhance sustainable management of rodent pests and subsequent control of rodent-borne diseases in disadvantaged urban communities across LMICs.

## Methods

### Overview

This is an experimental exploratory descriptive pilot study, targeted at defining national guidelines, and a standardised rodents surveillance protocol, in addition to conducting an operational comprehensive rodent surveillance training and setup a standardised surveillance within the urban disadvantaged community of Over-the-Hill, Nassau, the Bahamas (25° 4′ 11.40″ N 77° 20′ 4.87″ W).

To do this, we simultaneously conducted parallel but integrated activities, specifically: (1) developed a standardised surveillance protocol adapted to the local environment, (2) conducted 8 days of theoretical and practical rodent surveillance training (7th–14th June 2022), and (3) 3 months of pilot rodent surveillance after the training (June–August 2022), respectively (see details of the three activities under “guidelines preparation”, “preparation of course materials” and “pilot surveillance study” below). Reports of the “Rodent Control Assessment Workshop” and “Environmental Health Advisor for the Bahamas Hurricane Dorian Response” conducted in April–May 2019 and October 2019 were instrumental in developing this training. The reports highlighted the strengths and weaknesses of the Department of Environmental Health Services (DEHS), which is the arm of the Ministry of Environment and Natural Resources that drives the management of rodent population in the Bahamas. These reports revealed that rodent surveillance in the Bahamas relies on a “top-down” approach that lacks data standardisation across monitored areas, subsequently impacting the accurate definition of rodent infestation, damage level and pathogens spillover in the given area. Based on this, we developed a situational analysis, developed an explicit guidelines for rodent surveillance, as well as a multi-sectoral integrated rodent surveillance protocol that was used during the training and pilot surveillance program (see below, the course preparation, design and surveillance for details).

#### Guidelines preparation

Before the training session per se, we developed an integrated pest management (IPM) guideline (Supplementary Annex I) to aid the standardisation of site-specific rodent management protocol with likely interventions in the Bahamas. Briefly, we carefully explained the phases of IPM as applicable to the Bahamas. In particular, basic biology and ecology of rodents; the planning phase consisting of: identification of rodent-associated problems and areas to be surveyed based on previous study results & direct or indirect reports of rodent sightings/activities, the definition of activities and resources required for the programs; methods of rodent infestation surveillance essentially based on modified exterior and interior Centers for Diseases Control and Prevention (CDC) survey form^38^, and including direct and indirect observation of rodent signs, a survey using community perception/sightings, track plates, bait consumption, chew cards and rodent trapping; definition of rodent threshold levels which establish the level of acceptable rodent infestation in and around the household; possible interventions such as sanitation improvement, habitat management, diversionary feeding, exclusion, scaring and usage of repellents, chemical control, mechanical and biological control; evaluation of results, notably the assessment of the effectiveness of the intervention; and plans for the continuity of the actions likewise adherence to bio-safety measures.

#### Course preparation, design and surveillance

The rodent surveillance training team consisted of multidisciplinary professionals drawn up from four institutions, namely: the DEHS, PAHO, WHO and Instituto de Saude Coletiva, Universidade Federal da Bahia, who collaboratively worked to execute the rodent surveillance training and the post-training pilot surveillance. These four institutions regularly organised weekly meetings for 8-weeks before the training to plan and shape the training program. Specifically, they extensively studied the previous reports and the findings of Awoniyi et al.^30^ which characterised the unsatisfactory top-down method used for evaluating rodent infestation in urban communities of the Bahamas. This method principally relied on reports of rodent sightings lasting less than or equal to 4 h per day, and the ineptness of the rodent control method (chemical application) used across disadvantaged community to develop innovative surveillance and likely control strategies. These baseline reports guided the identification of the relevant items to be included in the training module, the type of rodent surveillance and format, teaching method, study area-sample size, materials and documents to be used, appropriate training period and the profession/number of the audience (trainers) and community members to be invited and engaged. Following the request of the DEHS representatives and subsequent evaluation during the teams’ weekly meetings, it was agreed that the training should focus on the following topics: basic rodent biology; the economic impact of rodents on agriculture, health and household properties; rodent survey methods (track plate, rodent trapping and exterior & interior household survey); definition of the study area; geographical identification of households/sampling points; dataflow; interpretation of data and community collaborative mapping; and rodent management (in the framework of an Integrated Pest Management approach). These topics were delivered by seasoned experts during the rodent surveillance training. It was believed that the wide panel of training components would inspire the standardisation of rodent surveillance, maintenance of accurate and unified data, and provision of essential information during rodent management program that would facilitate a better understanding of the drivers of rodent proliferation and subsequent intervention proposal that is suitable for the Bahamas.

#### Preparation of course materials (theoretical and practical)

##### Course procedure

To evaluate the socio-environmental factors driving rodent proliferation, the training course described techniques that are available to assist DEHS staff, Ministry of Agriculture and Marine Resources, the Ministry of Health and other local communities/multi-sectoral bodies that are interested in rodent-related studies in setting up rodent surveillance, gathering information about local rodent infestation and evaluating possible factors that could influence rodent infestation in the environment. For this purpose, the training was structured into two components: theoretical and practical. During the theoretical component of the rodent surveillance training, revised topics requested by the DEHS representatives were discussed.

As a baseline material, we modified previous manuals such as the CDC for exterior and interior rodent survey^38^, the track plate protocol from Hacker et al.^40^, the snap trap protocol from Woodman et al.^39^ and the “Levantamento Rapido de Indices para A*edes aegypti*—LIRAa” guideline by Brazil^55^ to develop specific protocols that remedy the flaws identified in the previous protocol and are suitable for the Bahamas’ condition. These Bahamas-specific protocols are described in detail under “preparation of protocols” and include the following components: definition of sampling size; preparation of maps; preparation of databases; approaching residents; household survey protocol, track plate and snap trap protocols as well as a road map for community collaborative engagement. The protocols were also used to train the course participants about different rodent survey techniques such as exterior and interior household rodent inspection, track plate and snap trap application.

Briefly, the household survey protocol provided a means of obtaining information about rodent infestations and environmental factors influencing rodent proliferation in the study areas. The track plate (TP) and snap trap (ST) protocols provided proxies for infestation level, providing information such as the distribution and approximate abundance of rodents in the environment which are fundamental for environmental management^40^. Likewise, considering that community participation in collaborative mapping is a proven instrument for collating information about local problems, residents’ perception and likely social solutions^58^, these were additionally used to identify high-risk locations for rodent infestation in addition to the experimental findings.

#### Preparation of protocols

##### Definition of sampling size

To test the developed protocol using a pilot-surveillance, we adapted the LIRAa guideline originally developed for *Aedes aegypti*^55^ to estimate the appropriate sample size for the rodent pilot-surveillance activities. To facilitate field logistics and sampling point identification, we divided the study area into blocks (using distinct geographical features such as at least three intersecting streets, roads and other man-made boundaries that can be easily delineated on a map) and used a conditional stratified random selection to choose the blocks to be surveyed in a way that minimize the selected blocks from sharing boundary within the study area. We then relied on a systematic random sampling technique to select one of every other household (sampling point) for rodent surveillance in each of the selected blocks. Specifically, each study area contained between 4 and 9 blocks depending on the size of the area, and each block contained on average 18 households (sampling points).

Briefly, the LIRAa protocol recommends sampling at least 450 households (number of sampling points—“n”) for an area possessing between 2000 and 8100 households, with the LIRAa guideline offering a formula that can be used to correct for the recommended 450 households based on the specific number of households/properties in the study area. This formula is shown below and used for calculating the required sample size for this pilot study:

- Household sample size (n): ${\text{n}}=450\div 1+(\frac{450}{N})$ where N = number of households or properties in the area, in this case N ≈ 2,527 with 144 blocks (A) using QGIS Version 3.22.

The approximate number of households in a block:

(b) Average number of households in a block (B): B = number of households or properties in the area/number of blocks of area (N/A) = where our N = 2527 and A = 144.
(c) Number of blocks to be surveyed (Q): From the definition of the number of properties to be sampled, it was necessary to determine the “Q”.
  $${\text{Q}} = {\text{household sample size}}\left( {\text{n}} \right) \div {\text{average number of households in a block}}/{2}.$$
  Therefore $${\text{Q}}=n \div \frac{{\text{B}}}{2}$$
  $${\text{Q}}=381\div \frac{18}{2}=42.$$
  Note: the value “2” in the denominator corresponds to the recommendation of sampling 50% of the households/properties in the selected blocks.

All blocks and households selected for the survey were numbered during the inspection to facilitate easy identification.

##### Preparation of maps and database

**Figure 3:**
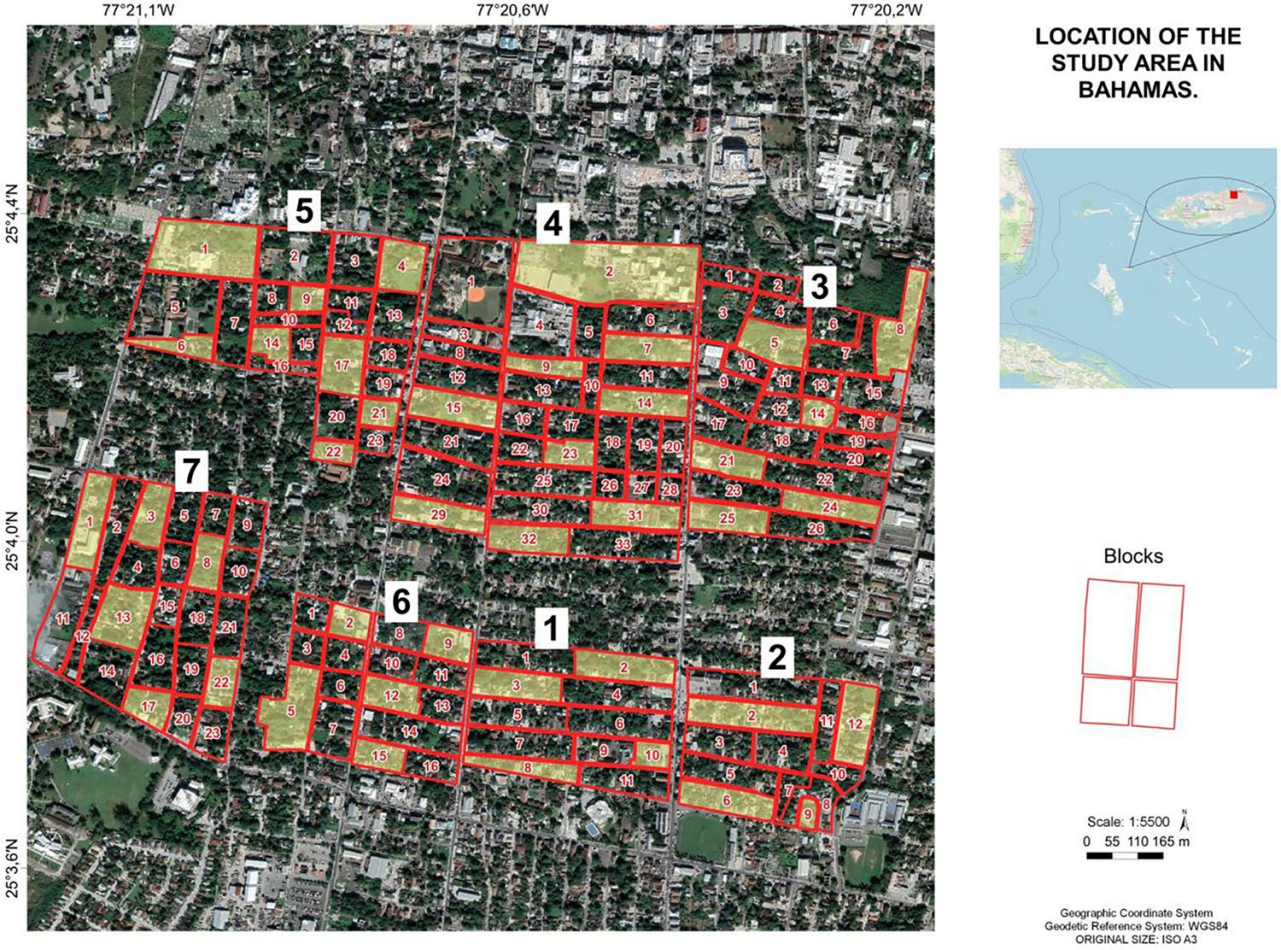
General study map showing the location of the blocks in the study community, blocks used for rodent surveillance are highlighted in yellow. The numbers 1 to 7 correspond to study areas 1 to 7. The figure was generated using QGIS Version 3.22 software.

Following the proliferation of rodents in the study areas after the occurrence of Hurricane Dorian in 2019, 50% of the affected areas (7 areas) were selected for pilot rodent surveillance as defined by the sampling strategy above. A map of the study areas containing the randomly selected blocks from each area was produced using spatial analysis software QGIS Version 3.22 (Fig. 3).

Additional maps containing the selected blocks from each study area were produced to assist the field teams in identifying the households to be visited and surveyed during the fieldwork. Figure 3 was also used during the community collaborative mapping to indicate possible rodent foci as identified by the residents.

To encourage data homogeneity and standardisation of results across study areas, we used web-based software called Research Electronic Data Capture (REDCap) to create a database for the project. The REDCap is a secured environment that can be used to store and export datasets into statistical programs such as SPSS, R, Stata, and Epiinfo, among other data analysis software (see Supplementary Annex IV for more details).

##### Approaching residents

We adapted the procedure of Pan-American Organization for Health^59^ to approach the residents upstream the field survey. Briefly, this protocol emphasised the need to: meet with community leaders and seek their approval for conducting a rodent survey; explain the details of the experiment i.e. its objectives, methods and expected benefits to the community; explain the required level of support from the community/residents; politely introduce the team members to residents; ask the residents if they could join in the survey of their residences and express appreciation for their collaboration while seeking the perfect opportunity to say goodbye after the inspection of their residences.

##### Household rodent survey

Households were surveyed to monitor and evaluate the severity of rodent infestation and the causal conditions encouraging rodent infestation in the area, in a way to guide future rodent intervention programs. We slightly modified the CDC^38^ manual to develop a simple household inspection procedure that consisted of observing active rodent signs (e.g. faeces, trails, grease marks, gnawed materials and active burrows) and identifying causal conditions (e.g. harbourage source-abandoned appliances, food source-improper waste disposal, animal feed, water source-leakages, stagnant water, etc.). We recorded and saved information obtained for each of these variables in the web-based REDCap software using a structured rodent exterior and interior survey form (Supplementary Annex II). The presentation of the team members and the project to the residents was followed by the inspection of residents’ facilities upon their approval at each surveillance point, and residents were encouraged to join in the inspection, if possible. Moving objects during the survey exercise was discouraged, while storage areas, kitchens and perimeters of the walls were thoroughly checked for any rodent signs (observed active rodent signs).

##### Ethics approval and consent to participate

We ensured that all methods were carried out in accordance with relevant guidelines and regulations, and reported in accordance with ARRIVE guidelines. Ethical clearance to conduct household rodent surveillance and trapping was granted by the Ministry of Environment and Natural Resources, Department of Environmental Health Services’ ethics committee. All participating residents whose houses were inspected during the environmental surveillance gave their informed consent before the survey, and consents were sought from residents aged 18 years and above.

The entire pilot study was conducted in compliance with the Bahamas laws regarding ethics in research.

##### Track Plates (TP) & Snap Traps (ST)

The protocols that have been extensively described and previously validated by Hacker et al.^40^ and Woodman et al.^39^ were simplified and used for TP & ST training, respectively. The TP is capable of evaluating the distribution and activities of rodents (but not their abundance or density), especially when carried out for two consecutive nights. A 0.2 × 0.2 m acetate sheet (TP) was painted with weather-resistant lampblack, which dried off in less than 5 min using a paint roller. The TP is capable of capturing different types of marks left by rodents, such as paw prints, tail marks and scratches. These marks were recorded and evaluated as positive signs of rodent infestation (see Supplementary Annex V-Part I for a brief guideline). The ST is useful for either managing rodent populations or for determining species richness and relative abundance. Field officers were encouraged to conduct at least one to two days of pre-baiting before trapping proper, as this has been shown to increase rodent trapping success and prevent neophobia among the rodent community^40,54,55,58–60^. Before trap placement, field officers obtained trapping permit or approval where necessary; checked the functionality of the traps; placed traps in areas with no or low livestock activities (to prevent the killing of non-target species); marked all trapping points on the map; placed traps in areas with active rodent signs (i.e. rodent runs, trails, burrows etc.); placed traps at noon and checked them at dawn while discarding carcasses according to the procedure for discarding biological waste (see Supplementary Annex V-Part II for a brief guideline).

##### Community collaborative meeting and mapping

The community conversation approach was planned and implemented based on years of study and experiences in vulnerable urban communities of Salvador, Brazil^61–63^. Residents belonging to different socioeconomic and education levels, age groups and genders, residing in the blocks of the study areas (with previous reports of rodent sightings) and who participated in the household rodent survey were deemed eligible and invited to the community meeting.

Members of the research team conducted visits and invited residents who live within the study community, presenting the objectives of the project and the organisations involved in the pilot study. The team also confirmed residents’ availability and communicated details (i.e. venue, day and time) of the meeting to the invitees. In total, 11 influential community members/participants, were selected to represent the study areas depending on its size (i.e. religious and civil society leaders who could express the community perspectives on rodent infestation, the causal factors and suggest strategic locations for potential intervention, while also assisting to disseminate the purpose and outcome of the meeting to other community members), during the community meeting and collaborative mapping. The meeting (face-to-face) was co-facilitated by a representative of the participants, ensuring a conducive environment for the free expression of opinions, with the participants entrusted with the responsibility of communicating the project initiative to other community members.

The meeting commenced with obtaining oral consent from e community member, followed by a brief self-introduction of both the participating community members and the research team. Subsequently, the purpose and outline of the program (Supplementary Annex VI-Part I) were presented to the participants. Using a semi-structured interview (Supplementary Annex VI-Part II), the dialogue primarily focused on subjects such as problems facing inhabitants of the community, knowledge about rodents, perception and exposure to risk factors, collaborative mapping, and actions to reduce risk, thus allowing participants to broadly express their minds even beyond the pre-conceived items contained in the training document. Upon participants’ responses, we provided suggestions on potential factors that could influence rodent circulation and control in the community, drawing from literature^2,6^ and previous studies from the same community^30^. For instance, participants were asked to consider the impact of unemployment, insecurity, proximity to abandoned property, extreme weather events, access to dumpsters and regular garbage pick-up schedule by the government, pets management, local method of rodent control, social custom/taboos about rodents and availability of resources required for rodent surveillance and control on the effectiveness of rodent population management. Each participant was allocated an average response time of between 15 and 20 min, resulting in over 3 h of conversation with the participants. The key opinions expressed by the participants were written by a member of the research team for evaluation during the expert meeting in an attempt to guide a holistic site-specific rodent surveillance and control program in the Bahamas. Key points from the minutes of the meeting were extracted and stratified into “factors capable of influencing rodent proliferation, suggested solutions and collaborative mapping of rodent-related issues at the area level” during the expert meeting. Although this method has faced criticism for perceived limitations in lacking rigor, it has been reported to provide locally specific in-depth information useful for guiding policymakers during decision-making^64^, and garnering local support for intervention initiatives^65^.

Also, during the community meeting, participants were also engaged in collaborative mapping (the process by which a group of individuals or community members work together to aggregate a map that reflects their collective knowledge, observations and experiences of a particular area or issue^58^), in this case, targeted at identifying areas or places with increased rodent presence; accumulated garbage; abandoned properties; accumulated rubble; and overgrown vegetation (Supplementary Annex VI-Part III). It is believed that this map would contribute to the definition of priority areas that require attention during intervention, and to promote future sustainable and culturally appropriate interventions based on community participation^58^.

#### Pilot surveillance study

##### Pilot community (over-the-hill)

The study community (Fig. 3) has been previously described in part by Awoniyi et al.^30^. The disadvantaged low-income community of Over-the-Hill was founded by freed African slaves in the 1800s and represented their core socio-cultural and educational activities post-slavery era. Over-the-Hill is bordered by School Lane to the north, Collins Wall to the east, Nassau Street to the west and Wulff Road to the south. However, the community that once produced the most accomplished and noteworthy Bahamians in modern history, has experienced a pitiful level of deterioration in recent years and is now a hotspot of violent and crime activities and poverty^66^. Over-the-Hill is the most marginalised and poorest community in the Bahamas. The community is now epitomised by dilapidated buildings with no access to electricity and potable water, overgrown properties, abandoned vehicles and furniture, haphazard dumping of refuse, frequent reports of rodent sightings, and high unemployment where only 5 out of every 100 working-age residents are often gainfully employed in formal jobs^66^. The current study community was chosen based on previous records of high rodent sightings^30^.

##### Surveillance points

Seven hundred and thirteen points were randomly selected across 42 blocks, as suggested in the definition of the sampling size for the cross-sectional survey. We excluded two hundred and fifty-six of the points due to inaccessibility or missing data, leaving us with four hundred and fifty-seven points. We used the household rodent survey form to extensively inspect the 457 points for three months (June–August 2022). Additionally, we conducted a-night rodent trapping at these points. Prior to the inspection, we followed recommendations on how to approach residents and carefully inspected designated households (as contained on the map) for active rodent signs. Following the surveillance exercise, all data obtained from the field (rodent surveillance and trapping) and community engagement meeting were uploaded to the REDCap account created earlier for subsequent analyses.

##### Analyses

To evaluate the socio-environmental variables associated with rodent proliferation in Over-the-Hill, we used a generalized linear model (GLM) with a logistic link and binomially distributed error structure. At each sampling point, our response variable was coded as 1 if there was at least one active sign of rodent infestation (such as reports of rodent sightings, runs, burrows, presence of faecal material, or gnawed material), and coded as 0 if none of these signs were noted.

Before testing for the effect of socio-environmental variables on rodent infestation (using active rodent signs as a proxy), we controlled for environmental variables that could influence rodent infestation. We first used separate GLMs to test for the relationships between the response variable and each of the following explanatory variables: premise type; presence of garbage, presence of water & food; the number of dwelling units; closeness to sewer, presence of unapproved refuse storage, privy closet, dilapidated fence and structural deficiencies; households with pipe or wiring gaps, presence of overgrown vegetation; the number of domestic animals; and harbourage access i.e. presence of construction materials. Variables with *p*-values of ≤ 0.15 from the single-factor models were included in a provisional multi-factor model since opting for the more conventional level of 0.05 at this stage could fail to identify all the important variables^67^. A mixed forward and backward stepwise model selection approach was then used to determine the final model using the Akaike Information Criterion (AIC). We chose the most parsimonious model with ΔAIC < 2 compared to the minimum as the final model^68^.

All analyses were performed in R version 4.3.0^69^, using the lme4 “nAGQ = 9”^70^ and MuMIn “v1.43.17”^71^ (Barton, 2020) packages.

### Supplementary Information

Supplementary Information 1.

Supplementary Information 2.

Supplementary Information 3.

Supplementary Information 4.

Supplementary Information 5.

Supplementary Information 6.

## Data Availability

All datasets and codes used during this pilot study are available in Zenodo under Creative Commons 4.0 license, accessible through https://doi.org/10.5281/zenodo.8023411.

## References

[1] Costa F (2014). Infections by *Leptospira interrogans*, Seoul virus, and *Bartonella* spp. among Norway rats (*Rattus norvegicus*) from the Urban slum environment in Brazil. Vector-Borne Zoonotic Diseases.

[2] Himsworth CG, Parsons KL, Feng AYT, Kerr T, Jardine CM, Patrick DM (2014). A mixed methods approach to exploring the relationship between Norway rat (*Rattus norvegicus*) abundance and features of the urban environment in an inner-city neighborhood of Vancouver, Canada. PLoS ONE.

[3] Lambert MS, Quy RJ, Smith RH, Cowan DP (2008). The effect of habitat management on home-range size and survival of rural Norway rat populations. J. Appl. Ecol..

[4] Meerburg, B. G. *et al*. Rodent-borne diseases and their risks for public health. 10.1080/10408410902989837 (2009). 10.1080/10408410902989837 19548807

[5] Belmain SR, Htwe NM, Kamal NQ, Singleton GR (2014). Estimating rodent losses to stored rice as a means to assess efficacy of rodent management. Wildlife Res..

[6] Masi E (2010). Socioeconomic and environmental risk factors for urban rodent infestation in Sao Paulo, Brazil. J. Pest. Sci..

[7] Murray MH (2021). “I don’t feel safe sitting in my own yard”: Chicago resident experiences with urban rats during a COVID-19 stay-at-home order. BMC Public Health.

[8] Byers KA, Lee MJ, Patrick DM, Himsworth CG (2019). Rats about town: A systematic review of rat movement in urban ecosystems. Front. Ecol. Evol..

[9] Dobigny G, Morand S (2022). Zoonotic emergence at the animal–environment–human interface: The forgotten urban socio-ecosystems. Peer Community J..

[10] Costa F (2014). Influence of household rat infestation on *Leptospira* transmission in the urban slum environment. PLoS Negl. Trop. Dis.

[11] Lam R, Byers KA, Himsworth CG (2018). SPECIAL REPORT: Beyond zoonosis: The mental health impacts of rat exposure on impoverished urban neighborhoods. J. Environ. Health..

[12] Aborode AT, Carla A, Mohan A, Goyal S, Rabiu AT (2021). Epidemic of plague amidst COVID-19 in Madagascar: Efforts, challenges, and recommendations. Trop. Med. Health.

[13] Yaro CA (2021). Infection pattern, case fatality rate and spread of Lassa virus in Nigeria. BMC Infectious Diseases..

[14] Knust B, Rollin PE (2013). Twenty-year summary of surveillance for human hantavirus infections, United States. Emerg. Infect. Diseases..

[15] Montoya-ruiz, C., Diaz, F. J. & Rodas, J. D. Recent evidence of hantavirus circulation in the American Tropic. 1274–1293 (2014). 10.3390/v6031274 10.3390/v6031274 PMC3970150 24638203

[16] Costa F (2015). Global morbidity and mortality of leptospirosis: A systematic review. PLoS Negl. Trop. Dis..

[17] Munoz-zanzi, C. *et al*. A systematic literature review of leptospirosis outbreaks worldwide *1970–2012*. 1–9. 10.26633/RPSP.2020.78 (2020). 10.26633/RPSP.2020.78 PMC7363284 32684917

[18] Allan KJ (2015). Epidemiology of leptospirosis in Africa: A systematic review of a neglected zoonosis and a paradigm for 'one health' in Africa. PLoS Negl. Trop. Dis..

[19] Colombe, S., Jancloes. M., Riviere, A. & Bertherat, E. A new approach to rodent control to better protect human health: First international meeting of experts under the auspices of WHO and the Pan American Health Organization. *WHO Weekly Epidemiological Record*, No. 17, 26 April 2019, (2019) https://apps.who.int/iris/bitstream/handle/10665/312103/WER9417-197-203.pdf?sequence=1&isAllowed=y Accessed 1 February 2023

[20] Byers KA (2019). “They’re always there”: Resident experiences of living with rats in a disadvantaged urban neighbourhood. BMC Public Health.

[21] Shah SN (2018). Housing quality and mental health: The association between pest infestation and depressive symptoms among public housing residents. J. Urban Health.

[22] Chelule PK, Mbentse A (2021). Rat infestation in Gauteng Province: Lived experiences of Kathlehong township residents. Int. J. Environ. Res. Public Health.

[23] De Klerk P, Van Dijk M, Van As AB (2016). Treatment and outcome of unusual animal bite injuries in young children. S. Afr. Med. J..

[24] Zahner GE, Kasl SV, White M, Will JC (1985). Psychological consequences of infestation of the dwelling unit. Am. J. Public Health.

[25] Rahelinirina S (2021). Rodent control to fight plague: Field assessment of methods based on rat density reduction. Integr. Zool..

[26] Taylor PJ, Arntzen L, Hayter M, Iles M, Frean J, Belmain S (2008). Understanding and managing sanitary risks due to rodent zoonoses in an African city: Beyond the Boston Model. Integr. Zool..

[27] Gratz, N. G. The role of WHO in the study and control of rodent-borne disease. *Vertebrate Pest Conference*. https://digitalcommons.unl.edu/vpc6/18 (1974).

[28] Lee MJ (2022). Municipal urban rat management policies and programming in seven cities in the United States of America. J. Urban Affairs..

[29] Dalecky, A. *et al*. Rodent proliferation in urban and agricultural settings of Sub-Saharan Africa—Part 2. Towards integrated management strategies, and beyond. *Preprints.org. *10.20944/preprints202301.0275.v2 (2023).

[30] Awoniyi AM (2021). Effect of chemical and sanitary intervention on rat sightings in urban communities of New Providence, the Bahamas. SN Appl. Sci..

[31] Pertile AC (2022). Evaluation of the impact of chemical control on the ecology of *Rattus norvegicus* of an urban community in Salvador, Brazil. PLoS ONE.

[32] Kaukeinen, D. E. Rodent control in practice: Householders, pest control operators, and municipal authorities. in (A. P. Buckle & R. H. Smith, Eds.) *Rodent Pests and Their Controls*, 249–271, (1994). CAB International

[33] Lambropoulos AS (1999). Rodent control in urban areas: An interdisciplinary approach. J. Environ. Health.

[34] Singleton GR, Leirs H, Hinds LA, Zhibin Z (1999). Ecologically-based management of rodent pests—Re-evaluating our approach to an old problem. Aust. Centre Int. Agric. Res. ACIAR Monogr..

[35] Swanepoel LH (2017). A systematic review of rodent pest research in Afro-Malagasy small-holder farming systems: Are we asking the right questions?. PLoS One..

[36] Third Global Leptospirosis Environmental Action Network (GLEAN) Meeting. GLEAN Meeting report, *Brasilia, Brazil,* March 12–March 14 2013. https://www.paho.org/hq/dmdocuments/2014/2013-CHA-Leptospirosis-GLEAN-Meeting-Report.pdf (2013).

[37] Sabatier P (1986). Top-down and bottom-up approaches to implementation research: A critical analysis and suggested synthesis. J. Public Policy.

[38] CDC. *Integrated Pest Management: Conducting Urban Rodent Surveys*. (2006)

[39] Woodman N, Timm RM, Slade NA, Doonan TJ (1996). Comparison of traps and baits for censusing small mammals in neotropical lowlands. J. Mammal..

[40] Hacker KP (2016). A comparative assessment of track plates to quantify fine scale variations in the relative abundance of Norway rats in urban slums. Urban Ecosyst..

[41] de Masi E, Pedro JV, Maria TP (2009). Evaluation on the effectiveness of actions for controlling infestation by rodents in Campo Limpo region, São Paulo Municipality, Brazil Access details: Access Details : [subscription number 913003116]. Int. J. Environ. Health Res..

[42] Panti-May JA (2016). A two-year ecological study of Norway rats (*Rattus norvegicus*) in a Brazilian Urban Slum. PLoS ONE.

[43] Richardson JL (2017). Using fine-scale spatial genetics of Norway rats to improve control efforts and reduce leptospirosis risk in urban slum environments. Evolut. Appl..

[44] Santos, N. D. J., Sousa, E., Reis, M. G., Ko, A. I. & Costa, F. Rat infestation associated with environmental deficiencies in an urban slum community with high risk of leptospirosis transmission. *33*(2), 1–13. 10.1590/0102-311X00132115 (2017). 10.1590/01021-311X00132115 28300969

[45] Costa F (2021). Household rat infestation in urban slum populations : Development and validation of a predictive score for leptospirosis. PLoS Neglected Trop. Diseases..

[46] Awoniyi AM, Venegas-Vargas C (2022). Population dynamics of synanthropic rodents after a chemical and infrastructural intervention in an urban low-income community. Sci. Rep..

[47] Dossou HJ (2022). Fine-scale prevalence and genetic diversity of urban small mammal-borne pathogenic Leptospira in Africa: A spatiotemporal survey within Cotonou, Benin. Zoonoses Public Health.

[48] Shafie NJ (2022). Prevalence of pathogenic Leptospira spp. in non-volant small mammals of Hutan Lipur Sekayu, Terengganu, Malaysia. Pathogens..

[49] Singleton, G. R. Ecologically-based rodent management integrating new developments in biotechnology. *Proceedings of the Vertebrate Pest Conference* 19. 10.5070/V419110207 (2000).

[50] Dobigny G (2018). Leptospirosis and extensive urbanization in West Africa: A neglected and underestimated threat?. Urban Sci..

[51] Schneider MC (2013). Leptospirosis: A silent epidemic disease. Int. J. Environ. Res. Public Health.

[52] Costa F, Martinez-Silveira MS, Hagan JE, Hartskeerl RA, Reis MGD, Ko AI (2012). Surveillance for leptospirosis in the Americas, 1996–2005: A review of data from ministries of health. Rev. Panam. Salud Pública.

[53] Rodrigues CM (2023). O círculo vicioso da negligência da leptospirose no Brasil. Rev Inst Adolfo Lutz. São Paulo..

[54] Awoniyi AM (2021). Using Rhodamine B to assess the movement of small mammals in an urban slum. Methods Ecol. Evolut..

[55] Brasil. Ministerio da Saude. Secretaria de Vigilancia em Saude. Departamento de Vigilancia das DoencasTransmissiveis–Brasilia. Levantamento Rapido de indices para *Aedes Aegypti* (LIRAa) para vigilancia entomologica do *Aedes aegypti* no Brasil: metodologia para avaliacao dos indices de Breteau e Predial e tipo de recipientes.84 p.ISBN 978-85-334-1999-5 (2013)

[56] Code of Practice (COP). COP for the prevention and control of rodent infestations on poultry farms. (2022). http://apha.defra.gov.uk/documents/surveillance/COP-rodent-infestations-on-poultry-farms.pdf

[57] Hawkins P, Golledge HDR (2018). The 9 to 5 rodent—Time for change? Scientific and animal welfare implications of circadian and light effects on laboratory mice and rats. J. Neurosci. Methods.

[58] Tavares, G. U. et al*.* Mapeamento colaborativo: uma interação entre cartografia e desenvolvimento sustentável no campus do PICI-Universidade Federal do Ceará. *Acta Geográfica,* 44–56, (2016)

[59] Organizacion Panamericana de la Salud (OPS). *Protocolos para la Vigilancia y Control de Roedores Sinantrópicos.* (2015)

[60] Gurnell J (1980). The effects of prebaiting live traps on catching woodland rodents. Acta Theriol..

[61] Hagan JE (2016). Spatiotemporal determinants of urban leptospirosis transmission: Four-year prospective cohort study of slum residents in Brazil. PLoS Neglected Trop. Diseases.

[62] Khalil H (2021). Poverty, sanitation, and * Leptospira* transmission pathways in residents from four Brazilian slums. PLoS Neglected Trop. Diseases..

[63] Reis RB (2008). Impact of environment and social gradient on *Leptospira* infection in urban slums. PLoS Neglected Trop. Diseases.

[64] Anderson C (2010). Presenting and evaluating qualitative research. Am. J. Pharm. Educ..

[65] Marisa BGC (1996). A ladder of community participation for underdeveloped countries. Habitat. Int..

[66] Hanna, R. All about Over-the-Hill, Overthehill Community Development Foundation. (2022) https://www.overthehillfoundation.org/community-challenges Accessed 25 January 2023

[67] Bursac, Z., Gauss, C. H., Williams, D. K. & Hosmer, D. W. Source code for biology and purposeful selection of variables in logistic regression. **8**, 1–8. (2008). 10.1186/1751-0473-3-17 10.1186/1751-0473-3-17 PMC2633005 19087314

[68] Burnham KP, Anderson DR (2002). Model Selection and Multimodel Inference: A Practical Information-Theoretic Approach.

[69] R Core Team. R: A language and environment for statistical computing. R Foundation for Statistical Computing, Vienna, Austria. URL (2019). https://www.R-project.org

[70] Bates D, Maechler M, Bolker B, Walker S (2015). Fitting linear mixed-effects models using lme4. J. Stat. Softw..

[71] Barton, K. MuMIn: Multi-Model Inference. R package version 1.43.17. https://CRAN.R-project.org/package=MuMIn (2020)

