# Supplementary material for: Systematic surveillance tools to reduce rodent pests in disadvantaged urban areas can empower communities and improve public health
Source: Sci Rep. 2024 Feb 24;14:4503. doi: 10.1038/s41598-024-55203-5 (PMC10894258; doi:10.1038/s41598-024-55203-5)
Supplement: Supplementary file 1 — Supplementary Information 1. [file 41598_2024_55203_MOESM1_ESM.pdf]

Systematic surveillance tools to reduce rodent pests in disadvantaged urban areas can empower communities and improve public health

Adedayo Michael Awoniyi[1,2†*], Ana Maria Barreto[2†], Hernan Dario Argibay[1], Juliet Oliveira Santana[3], Fabiana Almerinda G. Palma[1], Ana Riviere-Cinnamond[4], Gauthier Dobigny[5,6], Eric Bertherat[7], Luther Ferguson[8], Steven Belmain[9] & Federico Costa[1,2,3,10,11*]

[1]Instituto de Saúde Coletiva, Universidade Federal da Bahia, Salvador - BA, 40110-040, Brasil

[2]Instituto de Biologia, Universidade Federal da Bahia, Salvador - BA, 40170-115, Brasil

[3]Centro de Pesquisas Gonçalo Moniz, Fundação Oswaldo Cruz, Salvador Bahia, Brasil

[4]Data Management, Analytics and Products (DMAP), Health Information and Risk Assessment Unit (HIM), PAHO Health Emergencies, Washington DC USA

[5]French Institute of Research for Sustainable Development (IRD), UMR CBGP, Montpellier, France

[6] Pasteur Institute of Madagascar, Plague Unit, Antananarivo, Madagascar

[7]Department of Pandemic and Epidemic Diseases, World Health Organization WHO, Geneva, Switzerland

[8]Department of Environmental Health Services (DEHS), Ministry of Environment and Natural Resources, Government of The Bahamas

[9]Natural Resources Institute, University of Greenwich, Chatham Maritime, Kent ME4 4TB, UK

[10]Department of Epidemiology of Microbial Diseases, Yale School of Public Health, New Haven, CT06511, USA

[11]Lancaster Medical School, Lancaster University, Lancaster, LA1 4YW, UK

[†]These authors contributed equally and should be considered as co-first authors

*****Correspondence to: AMA | E-mail: maawoniyi13@gmail.com; FC | E-mail: federico.costa@ufba.br

ANNEX I- Bahamas IPM Guideline

Guideline for the Integrated Rodent Management (IRM) in New Providence, the Bahamas

This guideline is specially prepared for New Providence and the family islands of the Bahamas for regional rodent integrated pest management. The manual is also useful for classroom teaching, field training, non-governmental organizations (NGOs) interested in rodent management and others who work in community-based rodent integrated pest management.

Prepared by

Prof. Federico Costa

Dr. Adedayo Michael Awoniyi

2021

Table of Contents

Summary 4
Acronyms 5
1. Background 6
2.2 Phases of an integrated rodent management program for New Providence 13
  2.2.1 Planning 13
    2.2.1.1 Identification of the problem 13
  2.2.2 Definition of the area to be diagnosed 15
  2.2.3 Rodent infestation surveillance: 15
    Rodent infestation surveys 15
    Other rodent surveillance methods 16
  2.2.4 Definition of threshold levels 17
  2.2.5 Interventions 17
    Sanitation, Prevention and Control 18
  2.2.6 Evaluation of results 22
  2.2.7 Continuity of actions 23
2.3 Bio-safety measures 23
References 30
Annexes 35
    Annex 1: Rodent infestation surveys 35
    Survey units & personnel requirements and equipment 37
    Survey techniques (exterior & interior inspection) 38
    Annex 2: Other rodent surveillance methods 40
    Annex 3: Rodent control 42
    Annex 3A. Prevention and sanitation 42
    Annex 3B. Chemical control 43

Summary

Rodent populations are endemic in most cities around the world, partly due to human-engineered ecosystem changes in addition to other factors. ***The ability of rodents to adapt swiftly to all*** human-altered environments, their generalist eating behaviour and high reproduction rate are ***fundamental components in their propagation and management across the globe. Additionally, rodents' association with*** zoonotic pathogen transmission, infrastructural damage and consumption/contamination of food resources have placed them at the pinnacle of known pest species. Rodent control is a major public health measure that is implemented in most cities to reduce the risk of disease transmission, unfortunately most of the used methods have yielded mixed results. Thereby, ***the conceptualization of rodent control has opened up a new paradigm for location-specific integrated approaches. Therefore this protocol describes a careful explanation of the understanding of rodents' biology and ecology, and the need to accurately gather, analyze and interpret information obtained from the field, based on rodents' activities. Results from such information are crucial for developing appropriate guidelines that are necessary for successful location-specific intervention. Hence, this protocol that is Bahamas-specific, will aid the estimation of rodent infestations on the Island and characterize priorities areas that require intervention in order to manage rodent infestation and the associated rodent-borne diseases.***

Acronyms

CDC- Centre for Disease Control and Prevention

DEHS- Department of Environmental Health Services

EHAR - Environmental Health Advisor Report

GIS- Geographic Information System

KAP- knowledge, Attitude and Practices

IPM- Integrated Pest Management

IRM- Integrated Rodent Management

MMTV - The mouse mammary tumour virus

NGO- Non Governmental Organization

PAHO- Pan American Health Organization

PPE - Personal Protective Equipment

RCAW- Rodent Control Assessment and Workshop

US- United States of America

WHO- World Health Organization

1. Background

In the last two-five decades, the presence and abundance of rodents as well as the emergence and re-emergence of zoonotic diseases has been controlled by ecosystem changes in addition to other factors (Patz et al., 2004). Given the current unprecedented rate of global urbanization, there are concerns of the peri-urban environments rapidly becoming the hub of rodent population and rodent-borne infections, since these behavioural related changes promote the proliferation of rodents and subsequent frequent human-rodent interaction (Costa et al., 2014).

Synanthropic rodents account for about 40% of all mammal species and are found in all continents except Antarctica and all habitats except the ocean (Kay & Hoekstra, 2008). Rodents are capable of colonizing all human-altered environments, can reproduce rapidly especially in environments where there are abundant resources. Rodents are considered pests because they are reservoirs for several important viral, bacterial and parasitic diseases that have probably caused more human casualties than wars (Buckle & Smith, 2015; Costa et al., 2015). Likewise, they can destroy and contaminate agricultural products and infrastructure that is worth billions of dollars in a year (Battersby, 2015; Childs et al, 1991).

The Order Rodentia consists of more than 2,000 species of rodents, but just three are of utmost economic and medical importance. Namely *Rattus norvegicus*, *Rattus rattus* and *Mus musculus* that are referred to as commensal or synanthropic rodents because of their close association with human activities (Battersby et al., 2015).

The *R. norvegicus* (Norway or sewer rat)

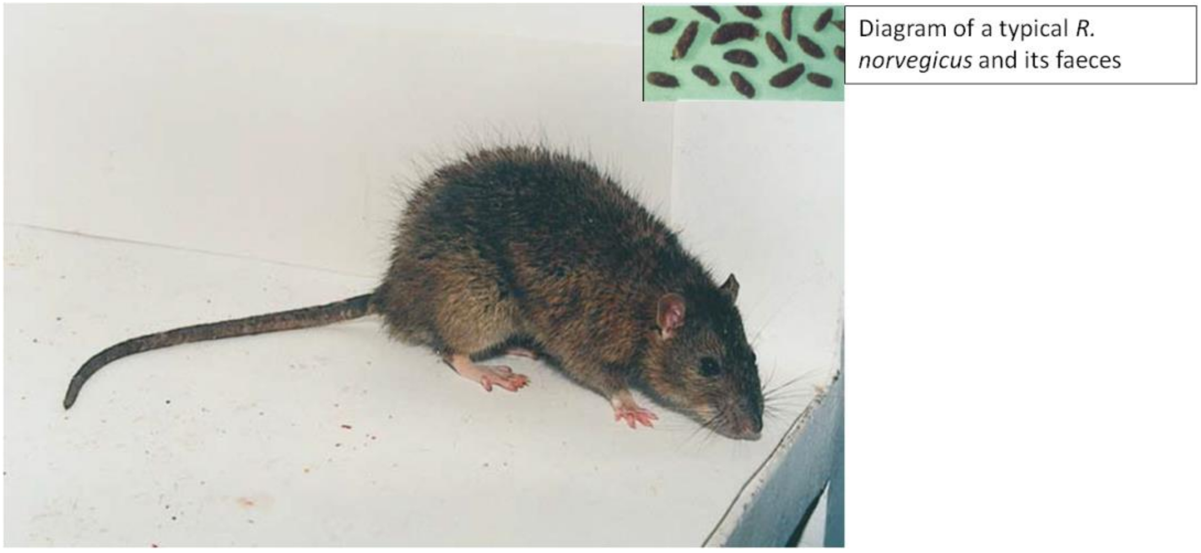

Diagram of a typical *R. norvegicus* and its faeces

Photo: Brazilian guideline for rodent control and surveillance

- *R. norvegicus* is found in six continents, and are well disposed to humid areas especially sewage or irrigated areas (Glass et al., 1989)
- Its invasion of landmass other than Europe and Asia coincided with the expansion of ship-based commerce accompanying the European colonization (Long, 2003)
- *R. norvegicus* is a major threat to endangered species on many colonized islands (Simberloff et al., 2013)
- *R. norvegicus* is well associated with an extensive list of zoonotic diseases (Meerburg et al.,2009)
- It is a generalist/opportunistic consumer, and prefers an underground burrow with a bed that is made from various materials
- Its total length is between 33-46cm, with body weight of between 110-480g
- Its body colour is greyish brown, grey or reddish brown and the eyes are small with a rounded snout
- Its tail is commonly shorter than the snout-to-anus length

***R. rattus* (Black, ship or roof rat)**

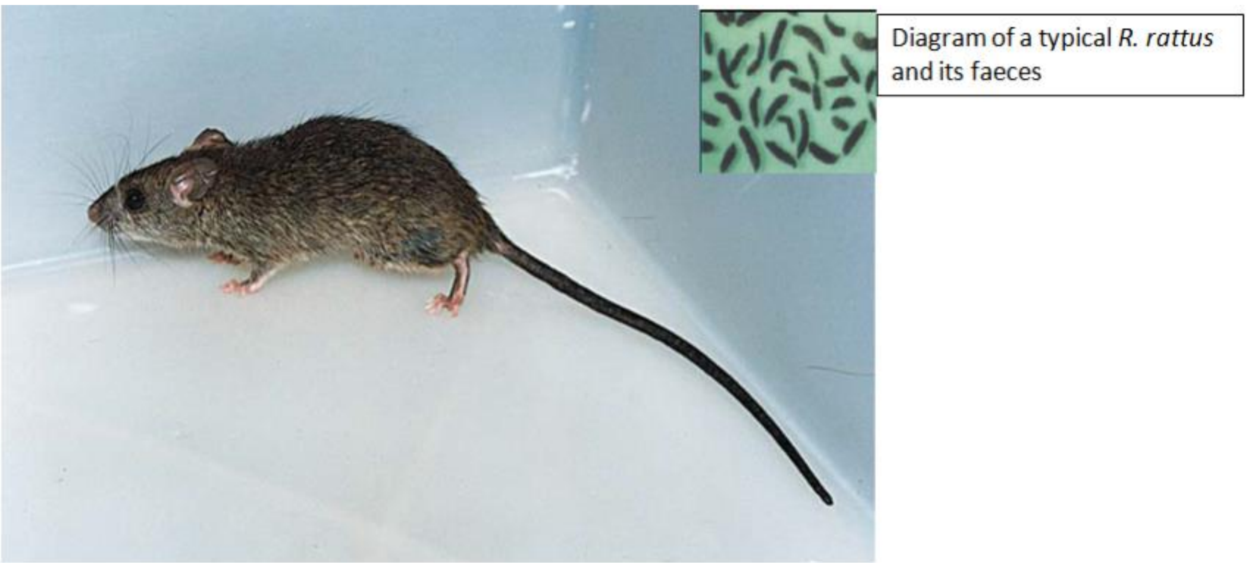

Diagram of a typical *R. rattus* and its faeces

Photo: Brazilian guideline for rodent control and surveillance

- *R rattus* originated from India and other Indo-Malayan nations and are commonly found in coastal areas because of its ability to flourish in human colonized areas as well as in large ship
- It serves as a reservoir of important zoonotic diseases and can inflict damage to agricultural produce (Nowak, 1999)
- *R. rattus* lives above the ground, and is also a generalist consumer, but consumes more of sprouts, roots, leaves, seeds and fruits
- It builds its nest above the ground usually on the trees, attics or walls from scraps of any material
- Its total length is between 35-45.5cm and body weight between 110-340g
- The body colour is black to brown while the eyes are large and prominent with a pointed snout
- The tail length usually exceeds the head-to-body length.

Considering the striking resemblance between *R. norvegicus* and *R. rattus* below chat provides quick supplementary distinguishing features between the two species.

Distinguishing characteristics that are useful in the identification of *R. norvegicus* & *R. rattus*

Adult rat	Roof rat<br>*Rattus rattus*	Norway rat<br>*Rattus norvegicus*
	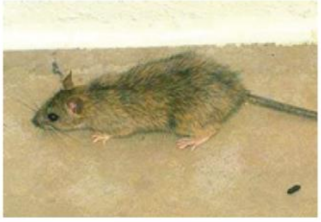	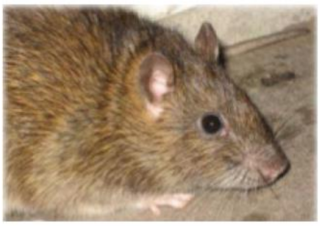
Adult head and body length in inches	7-8	8-10
Tail	Scaly, dark color. Nearly always longer than the body and head length.	Scaly, nearly hairless. Darker above, lighter underside. Shorter than head and body length.
Color	Variable from grey to brown to black, but most commonly smooth black hair with a lighter ventral underbelly.	Brownish/grey above, grey underbelly.
Ears	Large ears, almost hairless.	Small, covered with short hairs.
Snout	Narrow and pointed.	Blunt and bulky.
Droppings	Pointed, banana-shaped,<br>¼ - ½ inch long,<br>3/16 inch diameter	Blunt ends,<br>¾ - 1 inch long,<br>¼ - 3/8 inch diameter
Pairs of mammary glands	5	6
Nesting	May nest in high places, such as trees and attics, but they also can burrow. Maintain about a 120 square yard territory or less.	Construct nests in below-ground burrows or sometimes at ground level. Often line nests with paper, fabric, or other material. Burrow along building foundations, beneath rubbish or in dense shrubbery.

Chat adapted from the University of Arizona Cooperate Extension (Roof Rats Identification, Ecology & Signs by Paula Rivadeneira & Dawn H. Gouge)

***M. Musculus* (house mouse)**

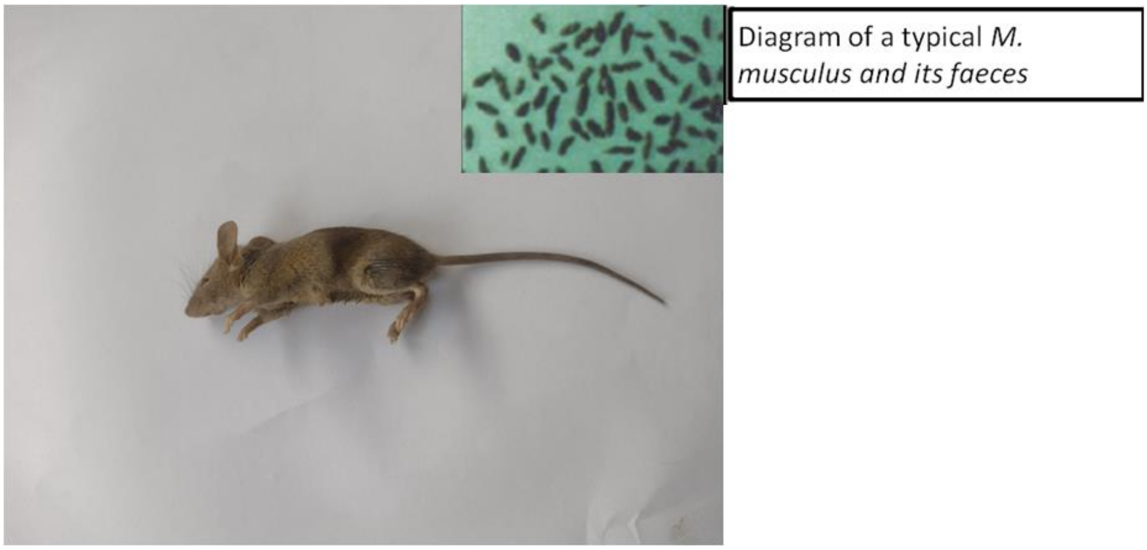

Diagram of a typical *M. musculus and its faeces*

Photo: Adedayo M. Awoniyi, personal archive

- *M. musculus* originated millions of years ago from the Indian subcontinent and comprises of subspecies that are defined based on geographical location (Boursot, 1993)
- It is capable of destroying human properties like furniture, books and clothing and can contribute to the spread of zoonotic diseases, notably of recent the transmission of virus called the mouse mammary tumour virus (MMTV) (Indik et al., 2005)
- Dwells in cracks or underground burrows consisting of a complex network of tunnels, several chambers for nesting and storage, with usually three to four exits in case of emergency when in the wild
- When dwelling with man, it nests behind rafters, woodpiles, storage areas or hidden spots near a food source
- Its total length is between 14.6-20cm and body weight between 13-26g
- The body colour ranges from light brown to dark greyish brown
- The eyes are black and small with a slightly pointed snout
- Its tail is short and usually half the total length in adult

The control of rodent population is an essential public health measure that is implemented in urban and peri-urban communities chiefly to reduce the risk of disease transmission (Lambert et al., 2008). In general, two methods are used for controlling rodents, namely chemical and non-chemical method, however both methods have yielded unsatisfactory long-term result. Therefore, an Integrated Pest Management (IPM) approach that proposes the combination of two or more chemical and non-chemical techniques (e.g., environmental education, code enforcement, rodent proofing, poisoning etc) is crucial to any rodent control program.

Therefore, this guideline aims to design a region-based procedure for quantifying rat infestation, while also proposing management procedures for the synanthropic rodent populations in the Bahamas. This guideline will expose field officers to the techniques necessary for gathering information about rodent infestation (surveillance), conditions favouring infestation (problem's root cause), and probable specific control measure (intervention) that is most suitable for their region.

2. Integrated Rodent Management (IRM)

This guideline provides the description and principles for basic methods of IRM. The principles are crucial, underpin the sustainable management techniques of pest and should be preserved in national pest control.

For more than half a century, the principles of IPM and specifically IRM have been successfully used worldwide. Developing an IPM/IRM program is more expensive and time consuming at first. However, the success of such programs has been extensively documented when compared to rodenticide-based strategies. While IRM is continually incorporating novel information and technology, its cornerstone however remains the same: restrict pests' access to food, water, and shelter and the pest will be controlled.

IPM is a practical strategy to pest control. Long-term pest management can be achieved by employing a mosaic of control strategies, such as:

- Public education,
- Improved sanitation,
- Rodent exclusion,
- Biological control techniques, and
- Mechanical control methods

IPM utilizes integrative approaches that manage pests and disease vectors through the environment in which the pests are most active.

In planning IPM, the

- Behaviour (birth rate, movement & population dynamics),
- Ecology (relationship with the environment) of the target species,
- Environment where the pest is active,
- Human behaviour towards rodents,
- Safety of the residents, likewise the non-target species must be duly put into consideration.

Usually, the utmost target of any IPM/IRM program is to minimize human pests encounter as well as to reduce pesticide exposure.

2.2 Phases of an integrated rodent management program for New Providence

There are some components of IRM program that are necessary for an effective rodent management. These components include **planning**; **survey**; **definition of threshold levels**; **interventions**; and **evaluation**.

2.2.1 Planning

This component majorly deals with:

- The identification of rodent-associated problems,
- Their causes (which consist of various biotic and abiotic variables),
- The areas to be surveyed,
- Definition of activities and the resources needed for the programs, and
- The expected results

Additionally, the phase basically answers the question of what the program wants to achieve, how, where and when it wants to achieve it and the team (institutions, groups & people) that will be involved in the program likewise their duties and positions.

2.2.1.1 Identification of the problem: Identify and justify the rodent problem that needs to be addressed, either from the perspective of rodent abundance, as well as animal or human health impacts associated with rodent-borne diseases. This guideline focuses on three main rodent-related problems which were agreed upon by the PAHO/WHO Country Office for The Bahamas & Turks and Caicos Islands and the National Authorities in The Bahamas, represented by the Ministry of Environment and Housing's Department of Environmental Health Services (DEHS), during a two period consultancy program performed in April-May and October2019 (EHAR, Nov. 2019). However, this list is not definite and could change depending on the possibility of identifying new problems that are associated with rodents. The identified priority problems are:

a) High rat infestation and risk of rodent-borne diseases in vulnerable neighbourhoods: Previous studies (Awoniyi et al., 2021) and reports (EHAR, Nov. 2019; RCAW, June, 2019) have shown high *R. norvegicus* infestation levels in some communities in the Bahamas, especially in urban areas with low socioeconomic status and migrant villages

(names withheld to avoid stigmatization). These areas provide environmental conditions (for example, abandoned vehicles, improper collections of garbage outside the households, overcrowding, flooding, inadequate and or haphazard sewer system) that support rats proliferation, thereby promoting close contact between man and rodent and the subsequent transmission of zoonotic diseases with the potential of spreading to other parts of the Island (Awoniyi et al., 2021 and Oyedele et al., 2015).

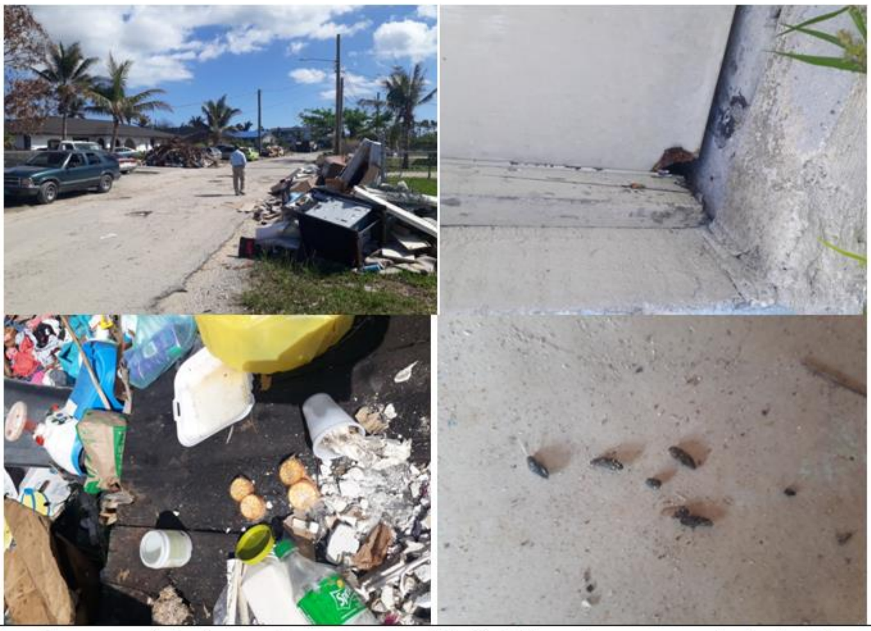

Plates showing some of the factors that are responsible for high rodent propagation in the Bahamas (i.e. indiscriminate refuse dumping; hole under the door; & unapproved human food storage)and an active rodent sign (faeces)
Photo: Federico Costa, personal archive

b) Potential rodent outbreaks and rodent borne disease outbreaks after natural disasters: With the ecological and geographical composition of the Bahamas and the projected sea-level rise and flooding (Strauss & Kulp, 2018), there are growing concerns about possible rodent proliferation and consequent likely rodent-borne disease outbreak on the Island, especially bearing in mind the likelihood of a rodent-borne disease outbreak following a natural disaster (EHAR, Nov. 2019). Previous studies have shown that natural disasters may change the mortality and spatial distribution patterns of rodents and consequent increase in pathogen prevalence (Long, 2003).

c) High rat infestation in tourist areas: Tourism is one of the driving forces of the Bahamas' economy. In the past, high rodent infestations were reported in some of the Bahamas' tourist areas (RCAW, June, 2019), (names withheld to avoid stigmatization)). This type of problem must be avoided given that the publicity about rat infestations

has been shown to negatively affect residents and tourists alike both physically and mentally (Parsons et al., 2020).

2.2.2 Definition of the area to be diagnosed: The boundaries of the area to be diagnosed must be determined. The main delimitation criteria will be based on environmental elements, and/or those derived from the socioeconomic and health characteristics of the community. Natural barriers such as streets with high vehicular circulation or bodies of water are good examples of such natural barriers, and the boundaries may change depending on survey activities. For example, an area initially included, but with evidence of lower rodent infestation during a recent survey, could be excluded from the intervention areas. Developing updated maps of the intervention areas is critical for operational activities. Furthermore, those maps will serve as baseline for the construction of risk maps after data collection.

2.2.3 Rodent infestation surveillance:

A critical component in assessing the role of urban rat population control is the use of reliable measures of rodent abundance. In this section we summarize the critical steps for the most frequently used method, the rodent infestation surveys. Also we list and explain other available methods potentially suitable for New Providence.

Rodent infestation surveys

Information about the degree of rodent infestation and associated environmental factors are generally obtained via

- Surveys and or monitoring community perception/rodent sightings/problem (Walsh, 2014; CDC, 2006)
- During the survey process, the presence of rats or rat signs (burrows, paths, droppings, fur & grease stains) is/are obtained by inspecting households and its surrounding premises i.e. residential, commercial, vacant lots and public areas in usually between 5-20% of the total area under consideration.

- Additionally, information about the socioeconomic status of the residents likewise the surrounding socio-environmental status are usually recorded.

Information collected during the survey is used to characterize infestation degrees at block and area levels, which is then summarized (the results) in tables, figures and maps. Results from this phase will provide information for the next two phases, definition of tolerance limit and identification of suitable interventions. Annex 1 provides additional information on urban rodent surveys guidelines, protocols and forms adapted from the CDC (2006).

Household interviews: This activity may be carried out in conjunction with the inspection to characterize residents' knowledge, attitude, and practices (KAP) about rodents and their acceptance of the different control alternatives. Given that human behaviour usually influences the effectiveness of rodent control, background knowledge obtained from residents will guide appropriate decision-making during control and prevention initiative (IPM). The relevant information may be used to generate percentage of positive responses per stratified unit (block or area) or complementary maps. The process for household interviews is also detailed in Annex 1.

Other rodent surveillance methods

There are other rodent surveillance methods including;

a) Rodent infestation surveys

b) Track plates

c) Bait consumption

d) Chew cards

e) Rodent trapping

f) Complaints

g) Rat sightings and several others.

A brief explanation on these methods and relevant literature are available in Annex 2.

2.2.4 Definition of threshold levels:

There exist a number of debates (Jacob & Halle, 2001; Burnham & Overton, 1978; Wiener & Smith, 1972; Tanaka, 1963) when it comes to defining an acceptable rodent tolerance limit within or around human dwellings. Considering the rodent surveillance methodology (Section 2.2.3), the CDC (2006) guideline defines threshold level of rodent populations and their causative conditions for defined target area as follow:

- a) 2% or less of the premises with active exterior rodent signs and either
- b) 15% or less of the premises with exposed garbage, or
- c) 30% or less of the premises with unapproved refuse storage.

These thresholds are based on previous studies of rodent control programs performed in heavily infested urban communities of the United States (US). While these thresholds are acceptable in cities of temperate developed countries, they may be too difficult to accomplish in urban tropical areas, especially in developing countries (Kogan & Bajwa, 1999). Despite the defined acceptable threshold limit for IPM rodent management in cities of temperate developed countries, there is a need for the local rodent IPM authorities to establish their own acceptable tolerance limit as necessary, as this will provide feedback for appropriately guiding rodent IPM program (Kogan & Bajwa, 1999).

- In indoor contexts, the ideal number or percentage of rodent within households should be zero, as man is not expected to live with rodents from the view of good public health practice (CDC, 2006).
- Therefore, the ideal tolerance limit for rodent population within or around human dwelling should be zero.

Establishing and sustaining a zero-tolerance limit for household rodent infestation requires effective management of factors that contribute to rodent dissemination, such as practicing appropriate waste management, and blocking all rodent entry points among others.

2.2.5 Interventions:

Among the IPM measures, the direct means of elimination of animals that is called "Control" stands out, and the indirect elimination method, or Prevention and

Sanitation are less attractive and more difficult to achieve. These two methods are not exclusive, on the contrary, they are complementary. However, all control should be accompanied by Prevention and Sanitation. In this context, the choice of the most appropriate control measure of an infestation must take into account the species of rodent, the level of infestation, as well as the location.

Sanitation, Prevention and Control

To lower rats' explosion, methods based on the installation of physical barriers and implementation of sanitation practices that is aimed at preventing rodents from having access to sources of food, water or shelter are used.

Basically, this constitutes:

- The process of improving local hygiene conditions, and
- Implementing structural and architectural changes to properties in a given location.

It is widely believed that for any rodent control program to be successful, it will be essential to improve the trash collection system and encourage proper collection and disposal of solid waste in a given location, in addition to the implementation of an adequate housing policy.

- **Notice** - Considering the current trash collection (twice/week) and waste disposal technique (indiscriminate dumping of waste) in the New Providence, there is an urgent need to upgrade the number of times that solid waste are collected to at least every other day.
- Alternatively, if the current waste collection regime is to be maintained, a standard waste management dumpster (~5 tons of 20ft * 8ft * 5ft dimension) should be installed in every block, with all households mandated to empty their solid waste there on daily basis

These measures are considered to have a collective effect, as they will impact large areas and benefit hundreds of people. However, within the concept of rats' population explosion, there are also individual measures, that is, those that each citizen can adopt in their property or place of work and leisure. These simple measures include;

- Daily disposal of garbage in the waste management dumpster or in the street a

few minutes before the pickup truck passes;

- Packing garbage in rat-resistant containers;
- Obstructing all existing gaps and crevices in the property with cement or sheet metal;
- Not leaving pet food on the floor; proper storage of food in places that are inaccessible to rodents;
- Eliminating debris, construction materials or abandoned objects that can provide shelter for rodents; among others.

Exclusion: this is a new paradigm in the pest management science that uses the selection of appropriate construction materials, and its application in the construction of barrier so as to reduce or eliminate rodent access into a facility (Corrigan, 2015). The method offers an effective long-term management for rodent population in an environment (Corrigan, 2015). Its strength depends on the ability to identify the active rodent signs and then apply the necessary action.

Habitat management: in some cases all that is needed to control rodent populations is the modification of their environments. Habitat modifications require curtailing rodents' access to food, water and harborage through the sealing of rodents' entry point or the usage of other barriers to block rodents' access into a facility.

Diversionary feeding: this term is often used interchangeably with supplementary feeding. This method uses food to divert the activities of rodents from an action that could result into negative impact (i.e. economic lost), although without the intention of encouraging their population expansion. This method has been employed in the management of wildlife impacts for example the case of forest damage by black bear (Ziegltrum, 2008). This method is often employed as a quick solution to a problem without much attention to longer term consequences of the target species (Blanco, 2006).

Scaring: this entails the use of scare tactics to divert the attention of pest away from a defined location. This method is achieved by the use of some novel materials in the defined location. For example, new sight, sound or odour which are all capable of scaring even man from a certain space.

Repellents: this method may be used to put off rodent populations, either by using the

natural or chemical-based repellents that are both commercially available and vary in effectiveness. The smells of some plants are offensive and unattractive to rodents however, this method is usually not ultimately effective in the absolute control or repelling of rodents.

Chemical control: studies have shown that the demographic structure of rodent populations can be altered by the removal of dominant individuals and the potential resulting immigration influx (Feng & Himsworth, 2014). Although other methods (non-chemical control) are also available for controlling rodents, however, results obtained from such are usually not satisfactory enough (Oyedele et al., 2015). Therefore, chemical control remains the backbone of any successful rodent control initiative (Buckle and Smith, 2015; de Masi et al., 2009). However, only chemical control seems not sufficient, as de Masi et al. (2009) in Sao Paulo, Brazil reported an initial 63.8% reduction in rodent infestation rate, and then a higher re-infestation rate of 79.8% six months after intervention.

Rodent control with first generation anticoagulant rodenticides: this generation of rodenticides requires that rodents consume the bait for several consecutive feeding periods for a lethal dose i.e. before a desired result can be obtained.

Tracking powders: these are often restricted class of rodenticides, and are majorly used by experts. Tracking powder is insoluble in water, and just a teaspoon full amount will control the population of Norway rats, Roof rats and House mice.

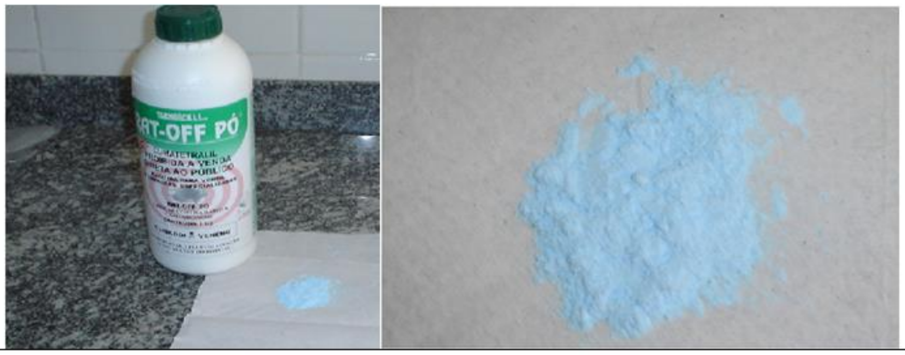

One of the first generation rodenticides that is commonly used against rats and mice
Photo: Federico Costa, personal archive

Saturation of pellets: this is also referred to as surplus baits, and this has to be made available to rodents throughout the control program.

Rodent control with 2nd generation anticoagulant rodenticides: this generation of rodenticides is more potent than the first generation, for example brodifacoum and it requires just a single dose to achieve a desired result.

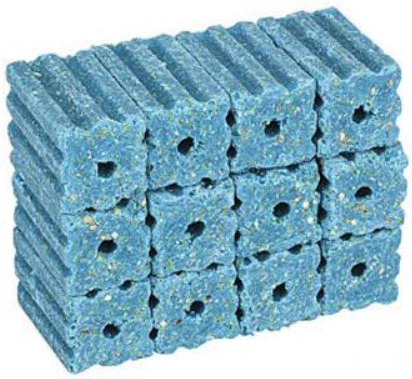

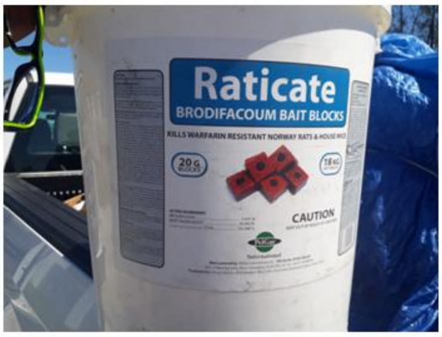

Common anti-coagulant rodenticides that are effective against rats and mice
Photo: Federico Costa, personal archive

Pulsed baiting: this is more toxic than the former, thus less quantity is required with less frequent toping up of the baits unlike saturation of pellet.

Mechanical control

Lethal traps: these are specialized rodent traps that are designed majorly to catch and kill rodents. They are usually set in an indoor location where there is suspected rodent activity.

Glue boards: these are trays that are coated with sticky adhesive, and are used to catch and kill rats and other crawling pests in a clean, economical and easy to use way.

Biological control

This is the process in which birds of prey; terrestrial predators such as cats and dogs in some cases; parasites; and bacteria are used as the main agents for the biological control of rodents. Example of bacteria that has been used is Salmonella, however none is rodent specific, therefore this control measure could cause severe infection in both man and animals (Wodzicki, 1973).

Fertility Control

This is the process by which contraceptives (plant extracts) are used to inhibit rodents' fertility either directly or indirectly (Tran & Hinds, 2013).

2.2.6 Evaluation of results

This aspect deals with the assessment of the effectiveness of the program. It determines whether results of the intervention meet the expected results, or if the interventions need to be repeated or modified. Normally, evaluation phase is done by scrutinizing selected specific indicators that signify and quantify useful information about changes in the situation that are considered problematic i.e.

- Rodent sightings,
- Presence of rodent signs,
- Source of food, water, among others.

As a result, a successful IPM intervention program should exhibit a reasonable level of efficiency before the end of the second week after the initiation of the intervention program (PAHO, 2015). These signs of effectiveness may be meassured by a variaty of metricss:

1. Signs of consumption of the bait in the case of chemical application method, is usually measured by the visit of the rodent to at least 50% of the baiting point
2. Signs of bait consuption is normally followed by at least 50% coloration in fresh rodent droppings, this coloration is as a result of the dye added to the bait by the manufacturers
3. At least 85% reduction in the number of active rodent signs in like three months post-commencement of the intervention program in the case of exterior survey
4. At least 15% or less in the total environmental factors sustaining rodent proliferation such as rodent access points, sources of water and food (CDC, 2006) in the case of interior survey, and
5. Trapping success (Coto, 2003)

2.2.7 Continuity of actions

In every IPM program, there can only be two basic outcomes which can then force two different responses

(a) The set objectives of the program are achieved: in this scenario, the program can proceed to its final stage (final report writing with conclusions and recommendations). In this case, a sustaining phase/user-friendly document is designed and the locals are trained and encouraged to continue leading practises that will sustain negative or low rodent population in their community.

(b) The set objectives of the program are not achieved: in this case, the program cycle is re-initiated. That is, the initial objectives of the program are re-evaluated mostly with minor/major modifications as the case may be and then re-implemented.

2.3 Bio-safety measures

Rodent controlling constitutes risks to the health of the employees involved in the program, the residents, as well as the non-target populations if not properly handled. Therefore, some essential preventive measures must be adopted:

a) **Safety measures during inspection** - Knowing that rodents are reservoir of various diseases, including leptospirosis.

   i. To inspect likely infested places, rodent control professionals must be careful not to step on or handle liquid collections without the use of proper protection (PPEs) of exposed body parts.

   ii. Control professionals must be aware of the possible risk of an object falling on them or other persons when moving furniture and objects.

   iii. When handling anything, you must protect yourself against sharp-edged surfaces

   iv. When entering properties, alleys, dependencies of public and private properties, pay attention to the presence of wild dogs or other animals that may attack.

v. Do not put your hand in holes or crevices. Try to use instrument/objects such as a long-handled tweezers (30cm) or a broom handle that are suitable for this purpose.

vi. Only enter private properties with the owner's authorization, or with legal support. Never jump over the wall or fence to access a property without a prior authorization of the owner.

vii. When you need to climb on roofs or walls, you must do so with the aid of a ladder (of appropriate height and make sure to securely fix it to avoid accident).

viii. Additionally, it is also advisable to use safety belts and an appropriate helmet when working at heights. When inspecting the freeboard and the bed of streams and rivers, a support stick should always be used, "to grope the bottom". A good tool for this is the broomstick. If the location to be inspected emits excessive noise, such as an engine room, for example, headset protectors should be used.

ix. You should also always pay attention to exposed electrical wires and cables that can cause electrocution, sometimes with fatal consequences.

x. Avoid stepping or walking on water or sewer pipes, as these could break.

xi. You must not eat, drink or smoke during inspection.

xii. Carcasses of animals or rodents found alive must not be handled without adequate protection.

b) **Safety measures in the application of rodenticides** - Every formulation of rodenticides constitutes risks to the health of the applicators, the population, especially children and the non-target animal species, who are at risk of direct ingestion of the product (primary poisoning) or from consuming contaminated/poisoned rodents and then becoming affected (secondary poisoning). Therefore:

i. Rodenticides application must take place in areas that are only accessible to rodents, hence, bait boxes or bait stations (available in the market) should be used in the outer perimeter of buildings or of fences

and arranged in contact with the walls duly labelled as “Rat Poison Please Keep Off/Rat Poison Please Do not Touch”.

ii. Place rodenticides indoors only when it is extremely necessary using bait boxes under the strict advice of experts, and in this case use only paraffin block formulations

iii. Rodenticides should not be used in places close to restaurants or food-handling centres, except when it is extremely necessary and under the strict supervision of experts

iv. The operator should not handle the baits with bare hands, e.g. when applying contact-powder formulation, applicators must use recommended PPEs

v. Leftover rodenticide baits must not be gathered together with other household wastes. They must be packed in plastic bags and taken to the operational base for proper disposal, according to the institution's rules

vi. To avoid rodenticides from being consumed by rodents that are originally assigned to other areas and consequently increasing the risk of accidents, granulated baits should be applied in bait holder boxes, waterproof blocks tied with wire to some structure of the place where they were applied.

vii. Also, tracking powder should only be sprinkled inside burrows.

c) **Safety Measures of the Applicator**: every professional directly involved in the application of rodenticides must use appropriate PPEs for handling the products and equipment (Figure 1).

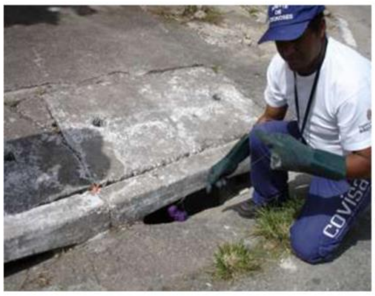

Figure 1: A rodent survey officer with his PPEs, i.e safety boot and glove to prevent direct contact with contaminated surface or specimen

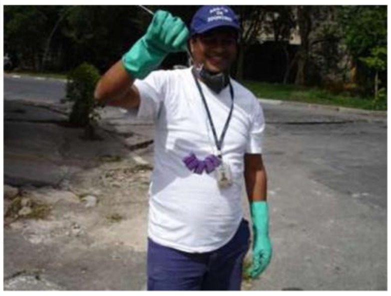

Avoid contact of the products with the skin and eyes. After the application, operators must sanitize or wash their hands, forearms and gloves with running water and soap. Never do this in kitchen sinks or cafeterias, rather use outdoor tanks or taps, e.g. laundry rooms. During transportation from the operational base to the field, rodenticides should not be transported to the field together with other chemicals e.g. insecticides as vapour from the insecticides could alter the palatability of the rodenticide. Likewise, when returning from the field, rodenticides and other equipment used in killing of rats must be transported in a separate compartment from the operators, for example, in the trunk of passenger vehicles or in the bucket of utility vehicles. Also, it is suitable for rodenticides to be carried inside closed plastic boxes (Figure 2).

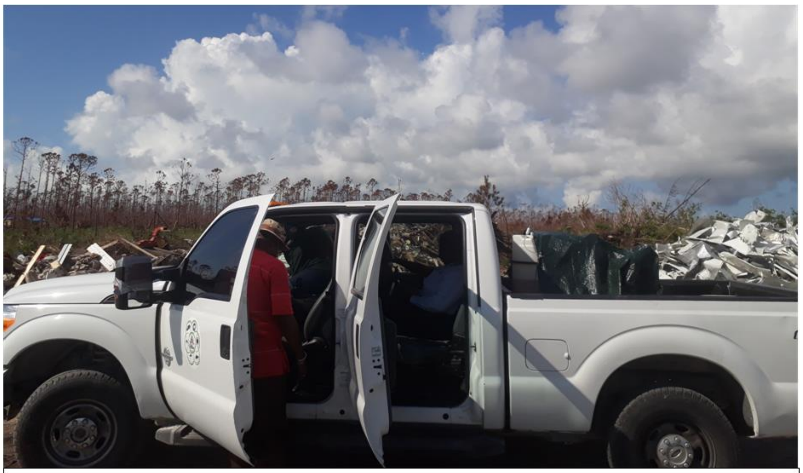

Figure 2: Example of a typical vehicle suitable for transporting rodent survey officers and necessary survey equipment

d) **Safety of the population**: Rodenticides must always be applied out of reach of children and/or curious people, or from the sight of passersby, if possible, rodenticides should not be applied in children schools or playground. When this is not possible, bait boxes should be used. Only rodenticides registered by the health regulatory agency should be applied. Never use products that are expired. Bait remains must be removed from the treated areas and disposed as chemical waste. Keep rodenticides in their original packaging, always closed, in a dry, ventilated place away from food. Do not apply rodenticides using mixed food and rodenticides, this is prohibited and do not contaminate drinking water sources with product residues.

e) **Safety of the non-target species**: make sure that other animal species including pets and the human residents do not have access to rodent baits or rodenticide and/or carcasses, so as to minimize the risk of unwanted poisoning. Do not use rodenticides against rodent species, or other wild animals without the prior authorization of the responsible agency. When applying rodenticides in open areas, such as gardens, squares or backyards, use bait boxes which must be identified with the details of the person responsible for the application and the product applied.

f) **Disposal of rodents killed by rodenticide**: immediately remove dead rodents during rats' explosion control. The following procedure must be observed during this process

i. Wear gloves when placing dead rodents in a double plastic bag
ii. Treat all carcasses as special waste, gather and deposit all carcasses in a designated biological waste bin that should only be handled by experts
iii. Decontaminate the underlying surfaces i.e. the soil where the dead rodents is recovered with 10,000 ppm sodium hypochlorite

Legislation

The national legislation on rodent control generally specifies the broad requirements of rodent control in homes and peri-domiciliary areas. However, the below legislation under the "Environmental Health Services Act" states some specific standards that are applicable to the Bahamas

Environmental Health Services Act

Part III – Prohibitions

9 (1) The owner or occupier of a premises is not permitted to allow conditions to exist which are unsanitary or constitute a nuisance that are conducive for the breeding or harbouring of rodents, insect pest, termites or other vermin.

Part V1. – Offenses and Penalties

20 (1) Any person who does not carry out or contravenes any provision made under the act is guilty of an offense. On summary conviction can be fined or imprisoned or both fine and imprisoned.

20 (2) An offender can be fined for each day that the offense continues after conviction.

Health Services - Health Rules

(Section 29)

Part I – Prevention and Abatement of Nuisances

5 (1) An Officer has the authority to conduct inspection of all premises

6 (1) Order of Abatement of Nuisances

When in the course of an inspection, an Officer finds unsanitary conditions or any breech of the Health Rules an order for abatement is serviced on the owner or occupier of the premises

8. Legal Proceedings

Rule 8 specifies how legal action is to proceed in the abatement

of

Nuisances

11 (1) Maintain Premises free garbage, etc.

The owner or occupier of premises is to maintain the premises free from any accumulation of rubbish, trash, kitchen refuse, garbage and disused containers by placing them in a bin for collection.

Part IV – The Production, Transportation, Handling Storage and Sale of Food and Drugs

68 Food establishments are required to be sanitary and free of ants, roaches, rats, mice and other vermin.

Food establishments are required to have a contract with a license exterminator as a licensing requirement

73 Disposal of Garbage

Garbage should be stored in a manner that it does not become a nuisance

Garbage should be stored in containers approved by the Director

Owners of business establishments are required to have a contract for collection of garbage with a license contractor

References

1. Arrieta, M., Rodolfo, S. Z., Rodolfo, G. R., Jaime, N. C., Carlos, H. M. & Julio M. C. (2001). Características de la población de roedores y pulgas en áreas de diferente riesgo para peste de tres provincias del departamento de Piura-Perú. Revista Peruana de Medicina Tropical y Salud Pública, 18 (3-4): 90-97.

2. Awoniyi, A. M., Thompson, A., Ferguson, L., Mckenzie, M., Souza, F. N., Zeppelini, C. G., & Costa, F. (2021). Effect of chemical and sanitary intervention on rat sightings in urban communities of New Providence , the Bahamas. *SN Applied Sciences*. https://doi.org/10.1007/s42452-021-04459-x

3. Battersby, S. (2015). Rodents as carriers of diseases. In Buckle, A., & Smith, R. (Eds.), *Rodent pests and their control* (2nd ed., pp. 81-101). *CABI International, Wallingford, Oxon UK* ISSN-13: 978 1 84593 817 8

4. Battersby, S., Hirschhorn, R.B., & Amman, B.R. (2008). Commensal rodents. In: Bonnefoy, X., Kampen, H., & Sweeney, K. eds. Public Health Significance of Urban Pests. World Health Organization, 387–421. Available at https://doi.org/10.1007/978-3-319-13884-8_31

5. Battersby, S., Hirschhorn, R. B., & Amman, B. R. (2015). Commensal rodents. In Buckle, A., & Smith, R. (Eds.), *Rodent pests and their control* (2nd ed., pp. 81-101). *CABI International, Wallingford, Oxon UK* ISSN-13: 978 1 84593 817 8

6. Blanco, G. (2006). Natural selection and the risks of artificial selection in the wild: nestling quality or quantity from supplementary feeding in the Spanish imperial eagle. Ardeola 53:341–351

7. Boursot, P., Auffray, J. C., Britton-Davidian, J., & Bonhomme, F. (1993). The evolution of the house mice. *Annual Review of Ecology and Systematics,* 24: 119-152

8. Buckle, A.P., & Eason, C.T. (2015). Rodent control methods: Chemical. In Buckle, A., & Smith, R. (Eds.), *Rodent pests and their control* (2nd ed., pp. 81-101). *CABI International, Wallingford, Oxon UK*. ISSN-13: 978 1 84593 817 8.

9. Buckle, A., & Smith, R. (2015). Rodent pests and their control: 2nd edition. In *Rodent Pests and Their Control: 2nd Edition*.

10. Burnham, K. P. & Overton, W. S. (1978). Estimation of the size of a closed population when capture probabilities vary among animals. Biometrika, 65 (3): 625-633.
11. Byers, K. A., Cox, S. M., Lam, R., & Himsworth, C. G. (2019). "They're always there": resident experiences of living with rats in a disadvantaged urban neighbourhood. *BMC Public Health,* 19(1): 853. https://doi.org/10.1186/s12889-019-7202-6
12. Caughley, G. & Sinclair, A. (1994). Wildlife Ecology and Management. Wiley-Blackwell. Cambridge.
13. Center for Disease Control - CDC. (1974). Urban rat surveys. Atlanta: US Department of Health, Education, and Welfare
14. Centre for Disease Control -CDC. (2006). Integrated pest management: conducting urban rodent surveys. Centre for Disease Control and Prevention- Atlanta: US Department of Health and Human Services
15. Childs, J. E., Glass, G. E., & Le Duc, J. W. (1991). Rodent sightings and contacts in an inner-city population of Baltimore, Maryland, USA. *Bull Soc Ecology,* 16: 245 – 255
16. Corrigan, R. M. (2015). "Of rodents and doors". PCT Magazine, GIE Media, Richfield, Ohio
17. Costa, F., Ribeiro, G. S., Felzemburgh, R. D. M., Santos, N., Reis, R. B., Santos, A. C., … Ko, A. I. (2014). Influence of Household Rat Infestation on Leptospira Transmission in the Urban Slum Environment. *PLoS Neglected Tropical Diseases*, *8*(12). https://doi.org/10.1371/journal.pntd.0003338
18. Costa, F., Wunder, E. A., de Oliveira, D., Bisht, V., Rodrigues, G., Reis, M. G., … Childs, J. E. (2015). Patterns in Leptospira shedding in Norway rats (Rattus norvegicus) from Brazilian slum communities at high risk of disease transmission. *PLoS Neglected Tropical Diseases*, *9*(6), 1–14. https://doi.org/10.1371/journal.pntd.0003819
19. Coto, H. (2003). Elementos distintivos de las ratas *Rattus rattus* y *Rattus norvegicus*. En: Manual de control de roedores en municipios. Fundación Mundo Sano. Buenos Aires.
20. de Masi Eduardo, Pedro José Vilaça, Maria Tereza Pepe Razzolini. (2009). Evaluation on the effectiveness of actions for controlling infestation by rodents in Campo Limpo region , São Paulo Municipality , Brazil Access details : Access

Details : [subscription number 913003116]. *International Journal of Environmental Health Research*, *19*(4), 291–304. https://doi.org/10.1080/09603120802592723

21. Dubock, A. C. (1982). Pulsed baiting. A new technique for high potency, slow acting rodenticides. In: Marsh, R. E. ed. *Proceedings of the Tenth Vertebrate Pest Conference*, 23-25 Februrary 1982, Monterey, California:123-136 (http://digitalcommons.unl.edu/cgi/view-content.cgi?article=1010&context=vpc10, accessed 15 February 2007)
22. Environmental Health Advisor Report - EHAR. (2019). The final report of the environmental health advisor for the Bahamas hurricane Dorian response. The Grand Bahamas: The Ministry of Environment and Housing's Department of Environmental Health Services (DEHS)
23. Eyre, M. T., Carvalho-Pereira, T. S. A., Souza, F. N., Khalil, H., Hacker, K. P., Serrano, S., … Giorgi, E. (2020). A multivariate geostatistical framework for combining multiple indices of abundance for disease vectors and reservoirs: A case study of rattiness in a low-income urban Brazilian community: A multivariate geostatistical framework for combining multiple ind. *Journal of the Royal Society Interface*, *17*(170), 1–21. https://doi.org/10.1098/rsif.2020.0398
24. Feng, A.Y.T., Himsworth, C.G. (2014).The secret life of the city rat: a review of the ecology of urban Norway and black rats (*Rattus norvegicus* and *Rattus rattus*). *Urban Ecosyst* **17,** 149–162, https://doi.org/10.1007/s11252-013-0305-4
25. Glass, G. E., Childs, J. E., Korch, G. W. & Leduc, D. E. (1989). Comparative ecology and social interactions of Norway rat (Rattus norvegicus) populations in Baltimore, Maryland. *Occasional Papers of the Museum of Natural History, University of Kansas, Lawrence, Kansas*. 130: 1-33
26. Gurnell, J. (1980). The effects of pre-baiting live traps on catching woodland rodents. *Acta Theriologica,* **25**:255–264. doi:10.4098/AT.arch.80-20
27. Hacker, K. P., Minter, A., Begon, M., Diggle, P. J., Serrano, S., Reis, M. G., … Costa, F. (2016). A comparative assessment of track plates to quantify fine scale variations in the relative abundance of Norway rats in urban slums. *Urban Ecosystems*, *19*(2), 561–575. https://doi.org/10.1007/s11252-015-0519-8
28. Indik, S., Gunzburg, W., Salmons, B. & Rouault, F. (2005). Mouse mammary tumour virus infects human cells. *Cancer Research,* 65(15): 6651-6659

29. Jacob, J. & Halle, S. (2001). The importance of land management for population parameters and spatial behaviour in common voles (Microtus arvalis). En: Advances in vertebrate pest management II. H. J. Peltz, D. P. Cowan y C. J. Feare (eds.). Filander Verlag Fuerth.
30. Kogan, M. & Bajwa, W. (1999). Integrated pest management: a global reality? *An. Soc. Entomol. Bras.* 28(1). https://doi.org/10.1590/S0301-80591999000100001
31. Lambert, M. S., Quy, R. J., Smith, R. H., & Cowan, D. P. (2008). The effect of habitat management on home-range size and survival of rural Norway rat populations. *Journal of Applied Ecology*, *45*(6), 1753–1761. https://doi.org/10.1111/j.1365-2664.2008.01543.x
32. Long, J. L. (2003). Introduced Mammals of the World: their History, Distribution and Abundance. CSIRO Publishing, Collingwood, Victoria, Australia, and CABI Publishing, Wallingford, United Kingdom. xxi + 589 pp. ISBN 0-643-06714-0
33. Meerburg, B. G., Singleton, G. R., & Leirs, H. (2009).The Year of the Rat ends—time to fight hunger! *Pest Management Science*, 65(4): 351-2
34. Mills, J. N., Childs, J. E., Ksiazek, T. G., Peters, C. J., & Velleca, W. M. (1995). Methods for trapping and sampling small mammals for virologic testing. U.S. Department of Health & Human Services. Retrieved from https://stacks.cdc.gov/view/cdc/11507
35. Modlinska, K., & Stryjek, R. (2016). Food Neophobia in Wild Rats (Rattus norvegicus) Inhabiting a Changeable Environment-A Field Study. *PLoS ONE* 11(6): e0156741. doi:10.1371/journal.pone.0156741
36. Murray, M.H., & Sanchez, C.A. (2021). Urban rat exposure to anticoagulant rodenticides and zoonotic infection risk. *Biol Lett*, 17: 20210311. https://doi.org/10.1098/rsbl.2021.0311
37. Nowak R. M. (1999). *Walker's Mammals of the World (6th Edition).* Baltimore, Mary land: Johns Hopkins University Press.
38. Oyedele, D. T., Sah, S. A. M., Kairuddin, L., & Ibrahim, W. M. M. W. (2015). Range Measurement and a Habitat Suitability Map for the Norway Rat in a Highly Developed Urban Environment. *Tropical Life Sciences Research,* 26(2): 27-44
39. Pan American Health Organization – PAHO (2015). Protocols for Surveillance and

Control of Synanthropic Rodent. Washington DC: Publication Program

40. Parsons, M. H., Kiyokawa, Y., Richardson, J. L., Stryjek, R., Byers, K. A., Himsworth, C. G., … Munch-South, J. (2020). Rats and the COVID-19 pandemic: Early data on the global emergence of rats in response to social distancing. *MedRxiv*, 2020.07.05.20146779. https://doi.org/10.1101/2020.07.05.20146779
41. Patz, J. A., Daszak, P., Tabor, G. M., Aguirre, A. A., Pearl, M., Epstein, J., Wolfe, N. D., Kilpatrick, A. M., Foufopoulos, J., Molyneux, D. & Bradley, D. (2004). Members of the Working Group on Land Use Change Disease Emergence. Unhealthy landscapes: policy recommendations on land use change and infectious disease emergence. *Environmental Health Perspectives*, 112: 1092-1098
42. Richards, C. G. J. (1989). The pest status of rodents in the United Kingdom. In: Putman, R.J. ed. Mammals as pests. London, Chapman & Hall Ltd: 21-33
43. Rodent Control Assessment and Workshop – RCAW. (2019). The final report of the DEHS on rodent control assessment and workshop on biology, surveillance and control of rodents. New Providence , the Bahamas: The Ministry of Environment and Housing's Department of Environmental Health Services (DEHS)
44. Simberloff, D., Martin, J. L., Genovesi, P., Maris, V., Wardle, D. A., Aronson, J. et al. (2013). Impacts of biological invasions: what's what and the way forward? *Trends in Ecology & Evolution,* 28(1): 58-66
45. Strauss, B. & Kulp, S. (2018). Sea-Level Rise Threats in the Caribbean: Data, tools, and analysis for a more resilient future. Retrieved from http://sealevel.climatecentral.org/uploads/ssrf/Sea-level-rise-threats-in-the-Caribbean.pdf
46. Tanaka, R. (1963). On the problem of trap response types of small mammals populations. Resources of Population Ecology, 5: 139-146.
47. Tran, T. T., Hinds, L. A. (2013). Fertility control of rodent pests: a review of the inhibitory effects of plant extracts on ovarian function. Pest Manag Sci. Mar;69(3):342-54. doi: 10.1002/ps.3354. Epub 2012 Jun 30. PMID: 22753347.
48. Twigg, G. (1975). The brown rat. Devon, David and Charles (Holdings) Ltd.
49. Sweetapple, P., & Nugent, G. (2011). Chew-track-cards: a multiple-species small detection device. *New Zealand Journal of Ecology,* **35**(2): 153-162 Available on-line at http://www.newzealandecology.org/nzje/

50. Walsh, G. M. (2014). Rat sightings in New York City are associated with neighborhood sociodemographics, housing characteristics, and proximity to open public space. *PeerJ* 2:e533;
51. Wiener, J. & smith, M. (1972). Relative efficiencies of four small mammals traps. Journal of Mammalogy, 53: 869-873.
52. Wodzicki, K. (1973). Prospects for biological control of rodent populations. Bull World Health Organ, 48(4):461-7. PMID: 4587482; PMCID: PMC2481104.
53. Woodman, N., Timm, R. M., Slade, N. A., & Doonan, T. J. (1996). Comparison of traps 487 and baits for censusing small mammals in neotropical lowlands. *Journal of Mammalogy,* 488 **77**(1): 274-281. https://doi.org/10.2307/1382728
54. Ziegltrum, G.J. (2008). Impacts of the black bear supplemental feeding program on ecology in western Washington. Human-*Wildlife Confl* 2:153-159

Annexes

Annex 1: Rodent infestation surveys

Organization of control and survey programs: this component seeks to characterize residents' knowledge, attitude and practices (KAP) about rodents and their acceptance of control initiatives. Given that human behaviour usually influences effective rodent control, background knowledge obtain from residents will guide appropriate decision-making during control and prevention initiatives (IRM).

Sample design and mapping: before the commencement of the fieldwork, it is advisable to organize a formal meeting with experienced statisticians to discuss the number of blocks/households that will be inspected and the acceptable margin of error. Upon concluding on the sample size, visit the field with GIS specialists to construct a clear map of the area specifying households to be surveyed.

Data collection design: before going to the field, establish the number and type of data to be collected, means of collecting the data (it is advisable to include at least one local in the team so as to aid acceptability by the community while also reducing risk of working in an unfamiliar environment). Also, the supervisor should occasionally go to the field with the field officers during data collection (quality control).

Construction of survey forms: in constructing survey form for IRM control and survey program, it is highly recommendable that the form contains the following seven basic components with each of the listed variables

1. **Demographics variables** - for example (a) number of inhabitants (b) household per capita income, US$/month
2. **Premise type and details** - (a) residential use only (b) commercial & residential (c) commercial (d) vacant lot (e) food vendor (f) premise located <20m to sewer/refuse (g) distance to open sewer/refuse (m) (h) borders an abandoned property
3. **Access to food & water sources** - (a) unapproved refuse storage (b) exposed garbage (c) animal food (d) fruit trees (e) open stores of human food (f) standing water (g) leaks
4. **Harbourage for rodents** - (a) abandoned vehicles (b) abandoned appliances (c) dilapidated fences or walls (d) plant related (e) exposed soil
5. **Entry/access of rodents** - (a) structural defects (b) pipe/wiring gaps (c) holes in the roof (d) holes on the walls (e) holes on the floor
6. **Signs of rodent infestation** - (a) rodent burrows (b) rodent runs (c) rodent faeces; *R. norvegicus*[a]*; R. rattus*[b]*; and M. Musculus*[c]
7. **Domestic animals** - (a) dogs (b) cats (c) chicken (d) others

[a]Capsule shaped and about 20mm long. [b]Ellipsoid shaped and about 12mm long. [c]Spindle shaped and about 6mm long.

<u>An example of CDC Urban rodent survey form (CDC 2006)</u>

City: Metropolis	Census Tract: 54-A	Inspector(s): H. Smith, A. Jones	Inspector(s) Initials: HS, AJ	Additional Block Information: 15 premises total accessed	Date	07	mm	26	dd	05	yy
County: Chandler	Block Number: 27				Page	1	of	2	Pages		

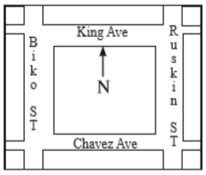

		Premises Type				Premises Details				Food				Water			Harborage							Entry/ Access		
No.	Premises Address	1. Residential	2. Commercial & Residential	3. Commercial	4. Vacant Lot	5. Food-Commercial	6. Vacant	7. No. of Dwelling Units	8. Sewers on Premises	9. Unapproved Refuse Storage	10. Exposed Garbage	11. Animal Food	12. Other Food & Plants	13. Standing Water	14. Condensate	15. Leaks	16. Abandoned Vehicles	17. Abandoned Appliances	18. Lumber/Clutter on Ground	19. Other Large Rubbish	20. Outbuildings/Privies	21. Board Fences & Walls	22. Plant-Related	23. Structural Deficiencies	24. Pipe/Wiring Gaps	25. Active Signs
1	646 Ruskin St.	✓						6	✓	✓	✓					✓					✓		✓	✓		✓
2	648 Ruskin St.	✓						4		✓	✓	✓	✓									✓		✓		
3	650 Ruskin St.				✓			0										✓	✓	✓					✓	✓
4	652 Ruskin St.	✓						8		✓	✓												✓			✓
5	654 Ruskin St.	✓						6		✓		✓										✓		✓		
6	[Chavez Ave.; data not shown]	—	—	—	—	—	—	—	—	—	—	—	—	—	—	—	—	—	—	—	—	—	—	—	—	—
7	661 Biko St.	✓						4		✓	✓			✓		✓					✓					✓
8	663 Biko St.	✓						3		✓	✓		✓	✓										✓		
9	[King St.; data not shown]	—	—	—	—	—	—	—	—	—	—	—	—	—	—	—	—	—	—	—	—	—	—	—	—	—
10	1243 King St.	✓						2		✓	✓											✓				✓
TOTAL		7	0	0	1	0	0	33	1	7	6	2	2	2	0	2	0	1	1	1	2	3	2	4	1	5

Recruitment and training of field officers: it is important to engage and train staff members who have utmost passion for IRM programs. During the training process, they should be taught how to correctly fill the survey form, interpret the objectives/questionnaire in clear terms, likewise the acceptable way of approaching the residents.

Analysis of data: collected information should be entered into an excel sheet and uploaded into the computer for an onward analysis using statistical tools like R, Stata, SPSS etc.

Survey units & personnel requirements and equipment

The followings are required during an IRM survey

a. Map of the area to be investigated with each premise clearly labelled
b. Survey forms/questionnaire
c. List of the premises to be investigated with contact details of the head of household
d. Writing materials
e. Backpacks
f. Digital camera/high ended telephone
g. Utility vehicle

The CDC (2006) manual can be used to calculate the size of the study area during an IRM survey in order to guarantee statistical power. A useful way of calculating the number of premises to be inspected is to divide the number of estimated premises by the number of blocks in the target area.

For example, $\frac{Number\ of\ estimated\ premises}{Number\ of\ blocks}$

In executing the survey program, at least two-man team is required for inspecting each block. During the survey, each team should be equipped with

a. field forms/questionnaire
b. writing materials
c. clipboard
d. hand gloves
e. backpack
f. plastic bags for field samples e.g. dead rodent, faecal materials e.t.c
g. insect repellent
h. digital cameras/ high ended telephone
i. mobile phone for communication between the supervisors and field teams especially in the case of emergency, and
j. black light to detect rodent urine stains and respirator for interior inspections.

Survey techniques (exterior & interior inspection)

The procedures for filling the forms/questionnaires likewise the questionnaires must comply with the CDC standard, so that information obtained from the program can be compared with those obtained from other countries/programs. Before setting out for the field, the following need to be done;

a) Field supervisor should inform head of households via different means of the proposed visit and the need for their utmost cooperation
b) He/she should also facilitate the grouping of trained field officers (minimum of two-man team per block)
c) Assign blocks among the teams (with each team planning its route before setting out of the office)

d) The field supervisor should be available to assist the teams if need be
e) If possible, field officers should commence their inspection at the northeast corner and then move clockwise
f) Of the two-man team, it is recommended that the most experienced officer handles the inspection while the other does the recording.

During inspection exercise, premises should be approached from the main entrance, with the interviewers presenting themselves neatly dressed in uniform (probably lab coat with identification card) during the entire program for safety purpose, and also to familiarize themselves with the community members.

The interviewers should take few minutes to courteously greet the head of the household, request a kind permission to conduct rodent inspection in their facility, and properly explain the purpose of the program to the responsible resident. Occupants of the facilities should be encouraged to join the team during the inspection, as this will in a way help to get their buy-in and aid the sustainability of the program.

The number of the premise should be boldly written on the form even before the commencement of the inspection, this will aid in reducing the number of missing data (NAs) during analysis, since information obtain on the field without an attachment to a particular household is as good as a blank or no information. After this, the team should proceed for interior inspection of the premises likewise its surroundings recording all structural or environmental deficiencies that may encourage rodent infestation or grant access into households on the survey form.

It is known that all animals usually leave signs of their presence in whatever environment they reside. These signs serve as evidence that such animals are active or reside in such environment even though they might not be seen physically at the time of surveillance. These indirect data are referred to as “active signs” and in the case of rodents “active rodent signs” such as footprints, burrows, paths, droppings, fur & grease stains. All these signs should be observed closely and recorded in the survey form. The inspector should inquire from the occupants where these signs could be seen and also conduct a thorough search in the yards, pet pens and food stores for these signs. Before vacating the premises, field officers should endeavour that all information are duly filled to limit the number of NAs during analysis.

In the case of interior inspection, the two officers should jointly check all rooms in the building for active rodent signs, particularly the kitchen, restrooms, food stores, and basement. Considering the ecotourism potential of the Bahamas, kitchens in bars, libraries, store houses, jetty stores, hotel facilities, shores of beaches and religion centres should be carefully inspected for any active rodent signs.

In situation whereby the resident rebuff the field officers from accessing their facilities for rodent inspection, the officer should note such premises and then contact the supervisor for appropriate action (maybe substitution with a nearby household or elimination from the survey in case the team has excess number of households)

Annex 2: Other rodent surveillance methods

Rodent trapping: Specific protocols have been published by CDC (2006) and Woodman et al., (1996). These documents describe the procedures for rodent trapping including identification of collection sites; trapping strategies; handling operation; and biosafety techniques (Mills et al., 1995).

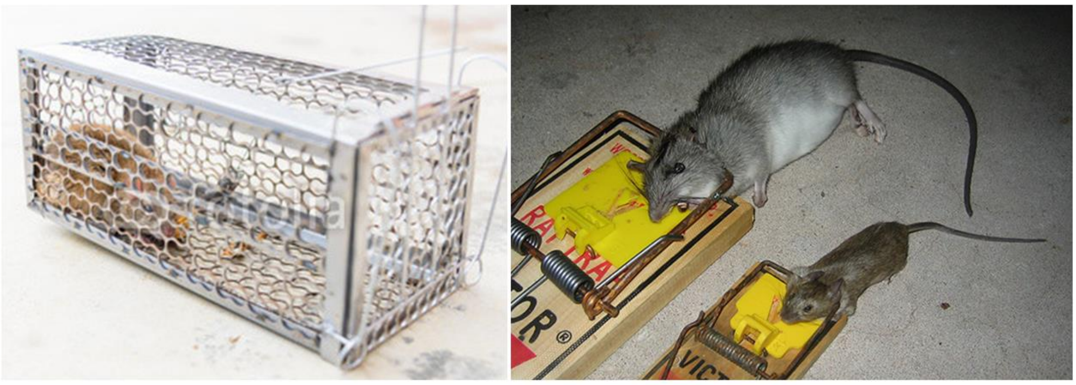

Example of a live trap and snap trap for rodent capturing

Photo: Fabio N. Souza & Adedayo M. Awoniyi personal archive

Track plates: This method has been previously described by Hacker et al. (2016) and Eyre et al. (2020) as a comprehensive proxy for evaluating the degree of rodent infestation in a particular area. The documents state the procedure for evaluating rodent infestation and then analyzing same.

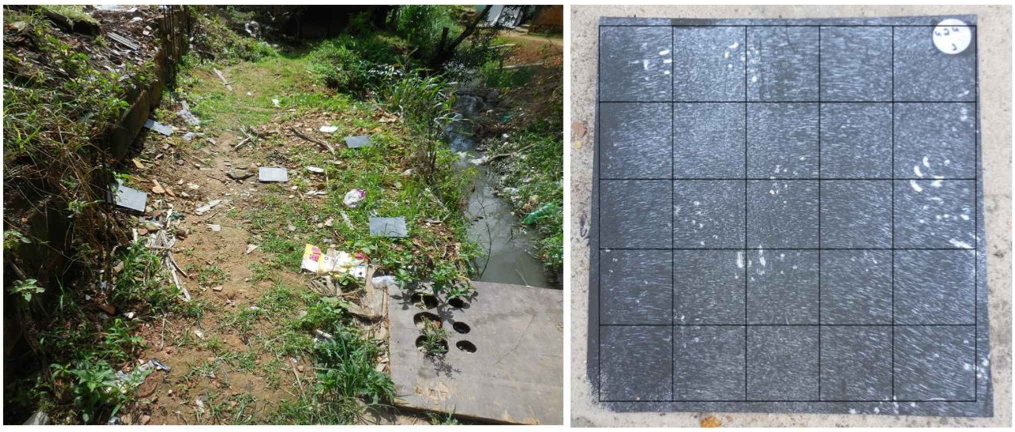

Positioning of tracking plate, with an enlarged positive plate

Photo: Fabio N. Souza personal archive

Bait consumption: Gurnell, J. (1980) has previously described the comprehensive protocol for rodent baiting. The document describes the procedures for rodent baiting, pre baiting and method of analyzing the level of bait consumption.

Chew track cards: these are made of corflute card filled with a lure that is attractive to the predators. The chew track card is a simple, new and cheap device that can be used to map the distribution and abundance of small mammals like rats, possums and hedgehogs over remote areas. The device record the animals' tooth and or footprints whenever they interact with the device, and the procedure for using this device has been extensively described by (Sweetapple & Nugent, 2011).

Complaints: Residents' complaints have been used previously as a means of evaluating household rodent infestation, and this has previously been described by (Parson et al., 2020; Richards, 1989). Reports are usually made when people file rodent-related complaints such as; active signs of rodents (grease stain, burrows, footprints, urine, droppings & gnawing), rat sightings and mouse sightings.

Rat sightings: Reports of rodent sightings by local residents or health/environmental officers have previously been used for evaluating household rodent infestation (Awoniyi et al., 2021; Childs et al., 1991; Walsh, 2014).

Annex 3: Rodent control

Annex 3A. Prevention and sanitation

Rodent prevention and control are one of the major public health measures taken in the urban communities, largely to reduce the menace of rodent sightings, rodent-human contacts and the risk of disease transmission (Lambert et al., 2008). Several factors are associated with rodent population bloom in any given environment, among which the availability of food, water and harbourage play an important role (Battersby et al., 2008; Costa et al., 2014). Therefore, adequate rodent prevention program of any sort would strongly rely on neutralizing the factors that support rodent population growth. For example, preventing rodents' access to food, water and harbourage sources, also sealing holes inside and outside homes would considerably aid the prevention of rodent infestation.

However, in situation where rodent prevention is rather too late, that is, when rat population is already established in a given location/environment, there exist several methods that can be used to control their onward propagation. Nevertheless, the choice of control strategy will depend on the type of premise; type of rodent; infestation level and presence of non-target species among others.

Amidst methods that are currently deployed for controlling rodent infestation is sanitation intervention, since deficient sewer systems is known to provide rodents with access to food, water and harbourage (Battersby et al., 2008; Costa et al., 2014), that in turn encourage the proliferation of rodents. To simply put, deficient sewer systems supply steady influx of food and water from garbage of all kind that are discharged into it; and reduce or control the population of rodent's predations in such environments (Battersby et al., 2008). Moreover, a heavily infested sewer can serve as a rodent-pool, that aid rodent repopulation even after a successful rodent control effort, thereby, deficient sewer system is the backbone of rodent population maintenance in any environment (Twigg, 1975).

Therefore, improved sewer system in addition to satisfactory sanitation program such as improvement of garbage collection and upgrading of open sewer system will

significantly support the control of rodent pest especially in urban environments (Twigg, 1975).

Annex 3B. Chemical control

Chemical application remains one of the easiest ways of tackling rodent population expansion, as its application is cheap, requires less technical know-how and less burdensome (Buckle & Eason, 2015). Also, the sole application of rodenticides offers immediate short-term effect on rodent population (Buckle & Smith, 2015; Lambert et al., 2008). Generally, there are two available generations (first and second generation) of chemical (anticoagulants/rodenticides) that are used for the management of rodent population.

A good example of the first-generation rodenticide is contact powder (cumatetralil). The contact powder is normally applied to the identified rodent trails and burrows, and it requires multiple exposures to function optimally. Contrarily, the second-generation rodenticide - paraffin block (brodifacoum), is usually applied to humid places, for example, sewers and leakages around houses and is more lethal than the first-generation, as a lethal dose could be ingested in a single feeding (Buckle & Eason2015). For both generations, applications should be carried out primarily in locations that are mainly accessible to rodents and not non-target species nor the residents.

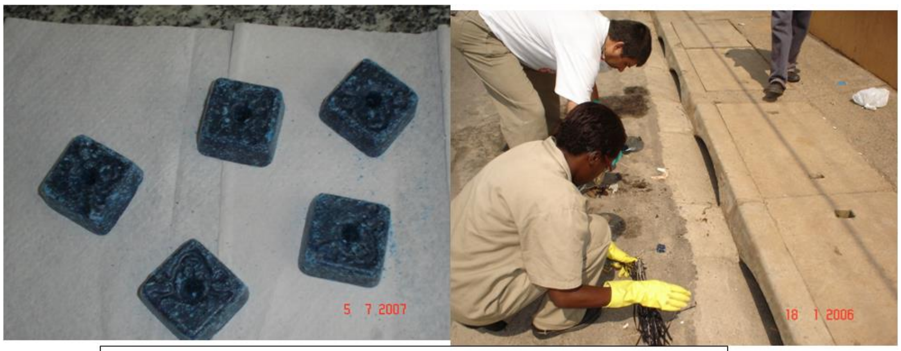

Rodenticide application by the field officers

Despite the popularity of rodenticides application among both the pest management authorities and local residents, it is not without its weaknesses.

1. Rodents could avoid rodenticide bait mixtures, a phenomenon that is referred to as neophobia that is, the act of avoiding new things which is common among rodents (Modlinska & Stryjek, 2016).
2. Rodenticides application has limited long-term success rate and could expose the surviving populations to higher chances of contracting and transmitting zoonoses (Murray & Sanchez, 2021)
3. Inappropriate usage of rodenticides could lead to rodents developing resistance toward the used rodenticides, the exclusion of non-target species or even emergence of impairment among residents
4. The handling of rodenticides by non-specialists could lead to selective killing, since feeding among rodents follow an hierarchical order (Dubock, 1982)
5. Foul odour could result from the use of rodenticides from dead rodent within residential areas, among others

Therefore, if effective rodenticides campaign is to be recorded, then it is important to develop possible ways of trouncing some if not all of the highlighted problems above. One way to go about this is the use of pre-baiting (Gurnell, 1980) to avoid neophobia among rodent community. Another method is the use of pulse baiting, that is, the deployment of a series of baiting rounds during which the baits are placed at a baiting station for a period of 3-13 days and then removed for a week, before the baits are replaced (Dubock, 1982). With this technique, the effect of hierarchical feeding that is common among rats is nullified, thereby allowing older rats to die before the bait is replaced, while also allowing non-dominant rats to also consume the bait. Pulse baiting also encourage bait conservation in that, dominant rats that have already consumed the bait do not have repeated excessive access to it before they eventually die. Finally if all the above stated steps are carefully observed that is, from problem diagnosis/survey to experimental design, execution and then evaluation of results (rodent survey and result analysis) which are the heart of IRM, then there will be little or no doubt that efficient rodent management would be achieved.
