# Supplementary material for: Systematic surveillance tools to reduce rodent pests in disadvantaged urban areas can empower communities and improve public health
Source: Sci Rep. 2024 Feb 24;14:4503. doi: 10.1038/s41598-024-55203-5 (PMC10894258; doi:10.1038/s41598-024-55203-5)
Supplement: Supplementary file 2 — Supplementary Information 2. [file 41598_2024_55203_MOESM2_ESM.pdf]

Systematic surveillance tools to reduce rodent pests in disadvantaged urban areas can empower communities and improve public health

Adedayo Michael Awoniyi[1,2†*], Ana Maria Barreto[2†], Hernan Dario Argibay[1], Juliet Oliveira Santana[3], Fabiana Almerinda G. Palma[1], Ana Riviere-Cinnamond[4], Gauthier Dobigny[5,6], Eric Bertherat[7], Luther Ferguson[8], Steven Belmain[9] & Federico Costa[1,2,3,10,11*]

[1]Instituto de Saúde Coletiva, Universidade Federal da Bahia, Salvador - BA, 40110-040, Brasil

[2]Instituto de Biologia, Universidade Federal da Bahia, Salvador - BA, 40170-115, Brasil

[3]Centro de Pesquisas Gonçalo Moniz, Fundação Oswaldo Cruz, Salvador Bahia, Brasil

[4]Data Management, Analytics and Products (DMAP), Health Information and Risk Assessment Unit (HIM), PAHO Health Emergencies, Washington DC USA

[5]French Institute of Research for Sustainable Development (IRD), UMR CBGP, Montpellier, France

[6] Pasteur Institute of Madagascar, Plague Unit, Antananarivo, Madagascar

[7]Department of Pandemic and Epidemic Diseases, World Health Organization WHO, Geneva, Switzerland

[8]Department of Environmental Health Services (DEHS), Ministry of Environment and Natural Resources, Government of The Bahamas

[9]Natural Resources Institute, University of Greenwich, Chatham Maritime, Kent ME4 4TB, UK

[10]Department of Epidemiology of Microbial Diseases, Yale School of Public Health, New Haven, CT06511, USA

[11]Lancaster Medical School, Lancaster University, Lancaster, LA1 4YW, UK

[†]These authors contributed equally and should be considered as co-first authors

***Correspondence to:** AMA | E-mail: maawoniyi13@gmail.com; FC | E-mail: federico.costa@ufba.br

ANNEX II: The rodent exterior and interior survey form

World Health Organization

WHO/PAHO Rodent Control Training
The Bahamas June 7-14, 2022

Pan American Health Organization

City: ______ Area: ___ Block: ______ House address: ________________<br>House condition: a. Inspected b. Closed c. Abandoned d. Deny access<br>Inspector(s) Initials __________ Date: ___/___/___		
**PREMISES TYPE**		
1. Premises type: a. Residential b. Commercial & Residential c. Commercial d. Vacant lot		
2. Food-Commercial:	**a.** Yes	**b.** No
3. No. of Dwelling Units:	**a.** Yes	**b.** No
4. Sewers on Premises:	**a.** Yes	**b.** No
**FOOD**		
5. Unapproved Refuse Storage:	**a.** Yes	**b.** No
6. Exposed Garbage:	**a.** Yes	**b.** No
7. Animal Food:	**a.** Yes	**b.** No
8. Other Food & Plants:	**a.** Yes	**b.** No
**WATER**		
9. Standing Water:	**a.** Yes	**b.** No
10. Condensate:	**a.** Yes	**b.** No
11. Leaks:	**a.** Yes	**b.** No
**HARBOURAGE**		
12. Abandoned Vehicles:	**a.** Yes	**b.** No
13. Abandoned Appliances:	**a.** Yes	**b.** No
14. Lumber/Clutter on Ground:	**a.** Yes	**b.** No

15. Other Large Rubbish:	**a.** Yes	**b.** No	
16. Outbuildings/Privies:	**a.** Yes	**b.** No	
17. Board Fences & Walls:	**a.** Yes	**b.** No	
18. Plant-Related:	**a.** Yes	**b.** No	
**ENTRY/ACCESS**			
19. Structural Deficiencies:	**a.** Yes	**b.** No	
20. Pipe/Wiring Gaps:	**a.** Yes	**b.** No	
**RODENT SIGNS**			
21. Active Signs:	**a.** Yes	**b.** No	*if yes*
21a. What type of signs:	Rodent Runs/Trails;<br>Rodent Faecal	Rodent Burrows;<br>Materials;	Others
21b. Species:	*Geocapromys ingrahami*;<br>*Rattus rattus;*	*Rattus norvegicus*;<br>*Mus musculus*;	Unknown
