# Supplementary material for: Systematic surveillance tools to reduce rodent pests in disadvantaged urban areas can empower communities and improve public health
Source: Sci Rep. 2024 Feb 24;14:4503. doi: 10.1038/s41598-024-55203-5 (PMC10894258; doi:10.1038/s41598-024-55203-5)
Supplement: Supplementary file 3 — Supplementary Information 3. [file 41598_2024_55203_MOESM3_ESM.pdf]

# Systematic surveillance tools to reduce rodent pests in disadvantaged urban areas can empower communities and improve public health

Adedayo Michael Awoniyi[1,2†*], Ana Maria Barreto[2†], Hernan Dario Argibay[1], Juliet Oliveira Santana[3], Fabiana Almerinda G. Palma[1], Ana Riviere-Cinnamond[4], Gauthier Dobigny[5,6], Eric Bertherat[7], Luther Ferguson[8], Steven Belmain[9] & Federico Costa[1,2,3,10,11*]

[1]Instituto de Saúde Coletiva, Universidade Federal da Bahia, Salvador - BA, 40110-040, Brasil

[2]Instituto de Biologia, Universidade Federal da Bahia, Salvador - BA, 40170-115, Brasil

[3]Centro de Pesquisas Gonçalo Moniz, Fundação Oswaldo Cruz, Salvador Bahia, Brasil

[4]Data Management, Analytics and Products (DMAP), Health Information and Risk Assessment Unit (HIM), PAHO Health Emergencies, Washington DC USA

[5]French Institute of Research for Sustainable Development (IRD), UMR CBGP, Montpellier, France

[6] Pasteur Institute of Madagascar, Plague Unit, Antananarivo, Madagascar

[7]Department of Pandemic and Epidemic Diseases, World Health Organization WHO, Geneva, Switzerland

[8]Department of Environmental Health Services (DEHS), Ministry of Environment and Natural Resources, Government of The Bahamas

[9]Natural Resources Institute, University of Greenwich, Chatham Maritime, Kent ME4 4TB, UK

[10]Department of Epidemiology of Microbial Diseases, Yale School of Public Health, New Haven, CT06511, USA

[11]Lancaster Medical School, Lancaster University, Lancaster, LA1 4YW, UK

[†]These authors contributed equally and should be considered as co-first authors

***Correspondence to:** AMA | E-mail: maawoniyi13@gmail.com; FC | E-mail: federico.costa@ufba.br

**ANNEX III: Variables used for evaluating rodent infestation across the study areas**

| Variables | Area 1 (n=57) | Area 2 (n=65) | Area 3 (n=67) | Area 4 (n=96) | Area 5 (n=59) | Area 6 (n=46) | Area 7 (n=67) |
|---|---|---|---|---|---|---|---|
| | n (%) | n (%) | n (%) | n (%) | n (%) | n (%) | n (%) |
| **Premise Type** | | | | | | | |
| Commercial food | 8 (14%) | 1 (1.5%) | 7 (10%) | 5 (5%) | 2 (3%) | 4 (9%) | 7 (10%) |
| Commercial | 9 (16%) | 9 (14%) | 22 (33%) | 37 (39%) | 16 (27%) | 12 (26%) | 13 (19%) |
| Commercial & Residential | 7 (12%) | 1 (1.5%) | 1 (1%) | 3 (3%) | 7 (12%) | 3 (7%) | 6 (9%) |
| Residential | 32 (56%) | 53 (81.5%) | 34 (51%) | 51 (53%) | 33 (56%) | 27 (58%) | 41 (61%) |
| **Vacant lot** | 1 (2%) | 1 (1.5%) | 3 (5%) | 0 (0%) | 1 (2%) | - | - |
| **Proximity to sewers** | 56 (98%) | 30 (46%) | 65 (97%) | 91 (95%) | 50 (85%) | 5 (11%) | 3 (4%) |
| **Residence with unapproved refuse storage** | 23 (40%) | 27 (42%) | 32 (48%) | 22 (23%) | 16 (27%) | 5 (11%) | 8 (12%) |
| **Exposed garbage** | 21 (37%) | 29 (45%) | 26 (39%) | 13 (14%) | 12 (20%) | 19 (41%) | 33 (49%) |
| **Animal food** | 12 (21%) | 25 (37%) | 14 (21%) | 9 (9%) | 7 (12%) | 11 (24%) | 8 (12%) |
| **Other sources of food** | 11 (19%) | 37 (57%) | 17 (25%) | 5 (5%) | 2 (3%) | 29 (63%) | 48 (71%) |
| **Standing water** | 3 (5%) | 15 (23%) | 5 (7%) | - | - | 2 (4%) | - |
| **Leaks** | 3 (5%) | 6 (9%) | 6 (9%) | 1 (1%) | 1 (2%) | 3 (7%) | 5 (7%) |
| **Abandoned vehicle** | 10 (18%) | 17 (25%) | 19 (28%) | 20 (21%) | 22 (37%) | 9 (20%) | 15 (22%) |
| **Bulk waste** | 11 (19%) | 35 (52%) | 20 (29%) | 12 (12%) | 9 (15%) | 20 (43%) | 35 (51%) |
| **Construction materials** | 18 (32%) | 36 (55%) | 29 (43%) | 16 (17%) | 6 (10%) | 26 (57%) | 40 (60%) |
| **Privies** | 17 (30%) | 6 (9%) | 15 (22%) | 13 (14%) | 7 (12%) | - | 2 (3%) |
| **Overgrown vegetation** | 24 (42%) | 19 (29%) | 21 (31%) | 15 (16%) | 20 (34%) | 18 (39%) | 32 (48%) |
| **Structural deficiencies** | 14 (25%) | 21 (32%) | 14 (21%) | 6 (6%) | 3 (5%) | 5 (11%) | 18 (27%) |
| **Pipe or wring gaps** | 8 (14%) | 14 (22%) | 10 (15%) | 1 (1%) | 1 (2%) | 8 (17%) | 17 (25%) |

NB: Boldened variables had p-value of ≤ 0.15 and were considered for the final model selection
