# Supplementary material for: Systematic surveillance tools to reduce rodent pests in disadvantaged urban areas can empower communities and improve public health
Source: Sci Rep. 2024 Feb 24;14:4503. doi: 10.1038/s41598-024-55203-5 (PMC10894258; doi:10.1038/s41598-024-55203-5)
Supplement: Supplementary file 4 — Supplementary Information 4. [file 41598_2024_55203_MOESM4_ESM.pdf]

Systematic surveillance tools to reduce rodent pests in disadvantaged urban areas can empower communities and improve public health

Adedayo Michael Awoniyi[1,2†*], Ana Maria Barreto[2†], Hernan Dario Argibay[1], Juliet Oliveira Santana[3], Fabiana Almerinda G. Palma[1], Ana Riviere-Cinnamond[4], Gauthier Dobigny[5,6], Eric Bertherat[7], Luther Ferguson[8], Steven Belmain[9] & Federico Costa[1,2,3,10,11*]

[1]Instituto de Saúde Coletiva, Universidade Federal da Bahia, Salvador - BA, 40110-040, Brasil

[2]Instituto de Biologia, Universidade Federal da Bahia, Salvador - BA, 40170-115, Brasil

[3]Centro de Pesquisas Gonçalo Moniz, Fundação Oswaldo Cruz, Salvador Bahia, Brasil

[4]Data Management, Analytics and Products (DMAP), Health Information and Risk Assessment Unit (HIM), PAHO Health Emergencies, Washington DC USA

[5]French Institute of Research for Sustainable Development (IRD), UMR CBGP, Montpellier, France

[6] Pasteur Institute of Madagascar, Plague Unit, Antananarivo, Madagascar

[7]Department of Pandemic and Epidemic Diseases, World Health Organization WHO, Geneva, Switzerland

[8]Department of Environmental Health Services (DEHS), Ministry of Environment and Natural Resources, Government of The Bahamas

[9]Natural Resources Institute, University of Greenwich, Chatham Maritime, Kent ME4 4TB, UK

[10]Department of Epidemiology of Microbial Diseases, Yale School of Public Health, New Haven, CT06511, USA

[11]Lancaster Medical School, Lancaster University, Lancaster, LA1 4YW, UK

[†]These authors contributed equally and should be considered as co-first authors

*****Correspondence to**:** AMA | E-mail: maawoniyi13@gmail.com; FC | E-mail: federico.costa@ufba.br

ANNEX IV- Preparation of database in REDCap

To encourage data homogeneity and standardisation of results across study areas, we used web-based software called Research Electronic Data Capture (REDCap) to create a database for the project. Descriptively, the REDCap uses instruments such as survey forms as research capture tools and offers a secure database that can be used for normal data entry or for surveys across multiple distinct times and points. The REDCap is generally designed to aid a secured environment that can be used by the research team to store and export datasets into statistical programs such as SPSS, R, Stata, and Epiinfo among other data analysis software. Additionally, REDCap offers more advantage over other web-based software in that it is quick and easy to operate; offers user-level access control (user-friendly); is accessible via mobile app (offline options); support both longitudinal and cross-sectional projects; provides the option to anonymously collect responses; fully customizable (provides the option to modify the database/survey questions at any time during the study); provides data comparison functions; offers access to researchers from various institutions (provided they have the login details); can save collected dataset in various format like pdf, csv, xlx etc. Therefore, we used the REDCap to create an entry for each variable that is contained in the rodent surveillance (exterior and interior) form and recorded such in the web-based REDCap software for further evaluation.
