# Supplementary material for: Systematic surveillance tools to reduce rodent pests in disadvantaged urban areas can empower communities and improve public health
Source: Sci Rep. 2024 Feb 24;14:4503. doi: 10.1038/s41598-024-55203-5 (PMC10894258; doi:10.1038/s41598-024-55203-5)
Supplement: Supplementary file 5 — Supplementary Information 5. [file 41598_2024_55203_MOESM5_ESM.pdf]

Systematic surveillance tools to reduce rodent pests in disadvantaged urban areas can empower communities and improve public health

Adedayo Michael Awoniyi[1,2†*], Ana Maria Barreto[2†], Hernan Dario Argibay[1], Juliet Oliveira Santana[3], Fabiana Almerinda G. Palma[1], Ana Riviere-Cinnamond[4], Gauthier Dobigny[5,6], Eric Bertherat[7], Luther Ferguson[8], Steven Belmain[9] & Federico Costa[1,2,3,10,11*]

[1]Instituto de Saúde Coletiva, Universidade Federal da Bahia, Salvador - BA, 40110-040, Brasil

[2]Instituto de Biologia, Universidade Federal da Bahia, Salvador - BA, 40170-115, Brasil

[3]Centro de Pesquisas Gonçalo Moniz, Fundação Oswaldo Cruz, Salvador Bahia, Brasil

[4]Data Management, Analytics and Products (DMAP), Health Information and Risk Assessment Unit (HIM), PAHO Health Emergencies, Washington DC USA

[5]French Institute of Research for Sustainable Development (IRD), UMR CBGP, Montpellier, France

[6] Pasteur Institute of Madagascar, Plague Unit, Antananarivo, Madagascar

[7]Department of Pandemic and Epidemic Diseases, World Health Organization WHO, Geneva, Switzerland

[8]Department of Environmental Health Services (DEHS), Ministry of Environment and Natural Resources, Government of The Bahamas

[9]Natural Resources Institute, University of Greenwich, Chatham Maritime, Kent ME4 4TB, UK

[10]Department of Epidemiology of Microbial Diseases, Yale School of Public Health, New Haven, CT06511, USA

[11]Lancaster Medical School, Lancaster University, Lancaster, LA1 4YW, UK

[†]These authors contributed equally and should be considered as co-first authors

[*]Correspondence to: AMA | E-mail: maawoniyi13@gmail.com; FC | E-mail: federico.costa@ufba.br

ANNEX V: Guidelines for Conducting Tracking Plate and Snap Trapping

WHO/PAHO Rodent Control Training
The Bahamas June 7-14, 2022

Part I- tracking plate

The principal objective of tracking plate

The main aim of conducting a tracking plate (TP) session is to specifically evaluate the distribution, abundance and activity of rats across the study locations.

Preparation of Lampblack Solution for TP activities

Materials Needed

- Lampblack powder
- 70% ethanol
- Laboratory Bottle
- Scale
- Hand gloves

Procedure:

- Measure 10 grams of lampblack powder into a tightly covered container
- Fill the container with 200mL of 70% ethanol and thoroughly shake the container to mix the solution
- After thoroughly mixing the solution, leave for at least 24hr before use

Painting of Tiles (plate) in preparation of placing TP in the field

Usually, we do conduct TP session for 2 consecutive nights, that is, TP will be painted on site, placed and examined the following day for two consecutive nights.

Materials Needed

- Lampblack solution
- 4” paint roller
- Spray bottle filled with 70% ethanol

- Tracking plates/boards
- Chalkboard
- Plastic bags

<u>Procedure</u>

- Place tracking plates/board on a chalkboard.
- Spray the paint roller and TP with ethanol
- Shake the lampblack solution
- Pour some amount of lampblack solution on the surface of the TP
- Rapidly use the paint roller to evenly coat TP with the lampblack solution.
- After painting, the TP tiles should be medium gray (image below)
- Allow tiles to dry for 5 min prior to placement

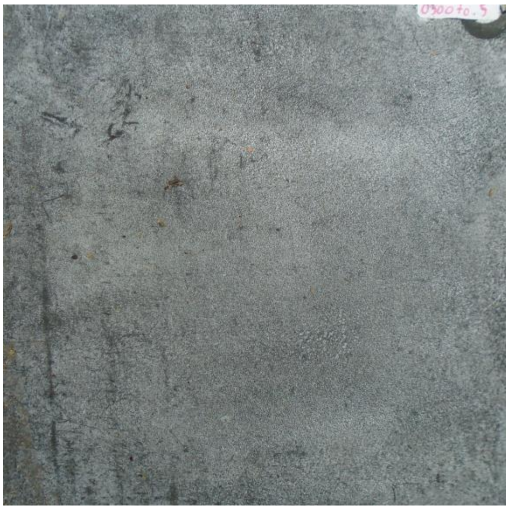

Example of a correctly painted TP

Placement of Tiles

<u>Materials Needed</u>

Painted TP tiles

<u>Procedure</u>

- 5 painted TP tiles
- Locate a 5m square free space for placing the painted TPs
- Place tiles in geometric shape with each tile 1m away from the rest, for example, the first tile should be positioned at the central point, while the rest 4 tiles should be placed 1m away from the 1 tile (see picture below).

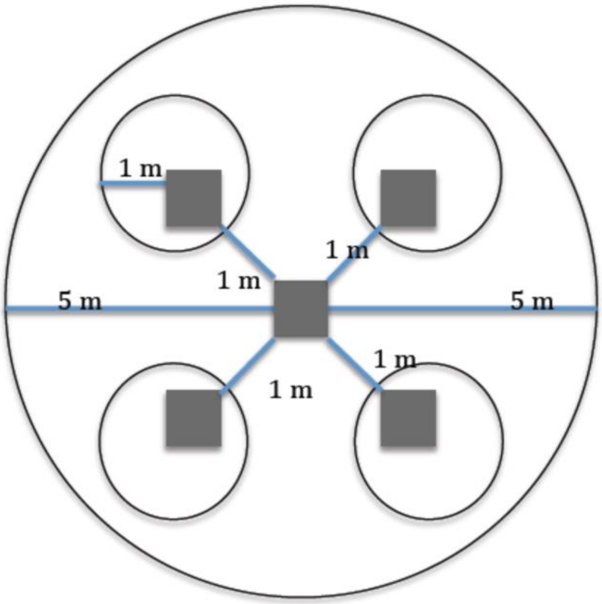

If the geometric shape cannot be maintained, as in very narrow areas, boards will be placed as shown below.

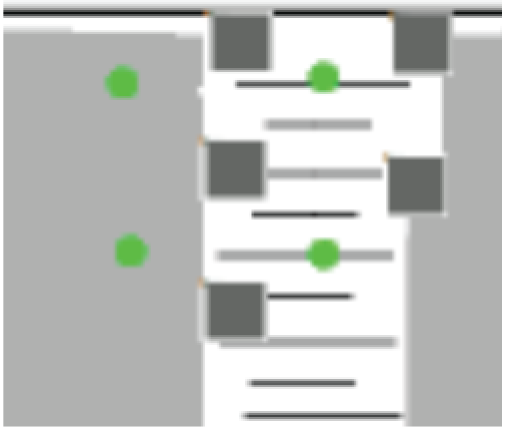

- Avoid placing TPs in a too opened areas, it is advised that TPs be placed along natural barriers, or near areas with active rat signs.

Photographs of TP tiles for evaluating the level of rat activity

Materials Needed

- Digital camera
- Chalkboard

Procedure

- Label each TP tile (tiles are labelled 1-5 plus the house/location ID on the bottom) before taken photograph
- Confirm that the tiles are correctly labelled.
- Arrange tile one chalkboard

- Take photographs of all present and unmoved TPs the morning after placement
- Take a neat picture directly over each tile, preferably under a shade.

Replacing and Repainting Tiles after first day of TP

Materials Needed

- 40 extra tiles
- Prepared Lampblack solution
- 4” paint roller
- Spray bottle filled with 70% ethanol
- Chalkboard

Procedure

- Following photographs of all present and unmoved tiles, all 5 tiles will be repainted (with lost tiles replaced).
- Follow the procedure above for the placement of tiles
- Then replaced tiles in the same position used the previously.
- Any missing tiles will be replaced with new painted tiles

Scoring and Analysis of Tiles to determine the amount of the grid with rodent mark

Materials Needed

- Computer
- Redcap data system
- PowerPoint presentation

Procedure

- Using PowerPoint, overlay 5 x 5 grid on tracking board pictures using a new PowerPoint for each tile each date
- Example of Grid Overlay:

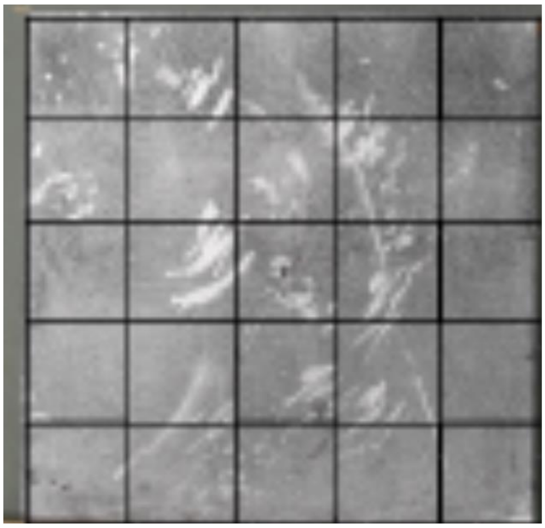

- Access the “Tile 1” or “Tile 2” (Tile 1 for day 1 TP activities, Tile 2 for day 2 TP activities) questionnaire on Redcap
- Fill out questionnaire

a. The number of grids (denominator) = the number of total grids (25) – the number of grids that need to be censored using the protocol bellow:

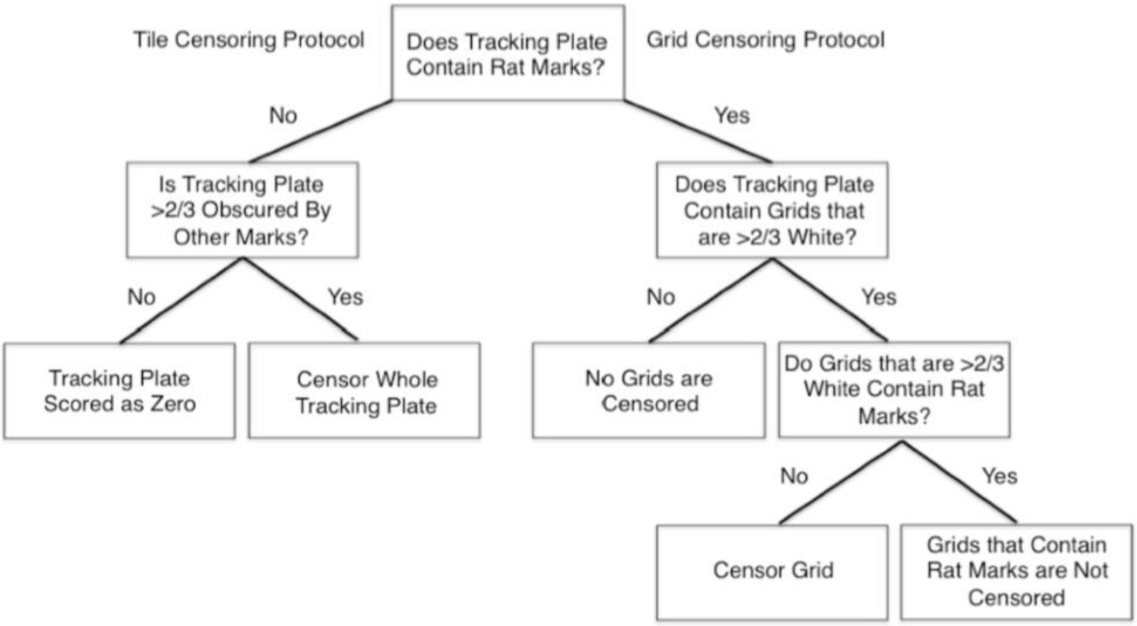

b) This will help to adjust for potential issues with confounding with other marks on the tiles. The number of grids that contain rat marks = The total number of grids that contain either paw prints, tail marks, or rat scratches. This creates an overall score of number of grids with rat marks/25

Examples of Rat Marks and other Common marks:

Types of markings observed on track plates.

- Rat paw prints
- Rat tail mark
- Rat scratches,
- Possum paw prints
- Dog paw prints
- Chicken foot mark
- Cat paw print
- Snake mark,
- Human shoe print
- TP left out in torrential rain storm.

Track plates that contained rat

Paw prints

Tail marks or rat scratches will be considered positive track plates (i.e. track plates with rat markings).

Common Rat Marks Observed

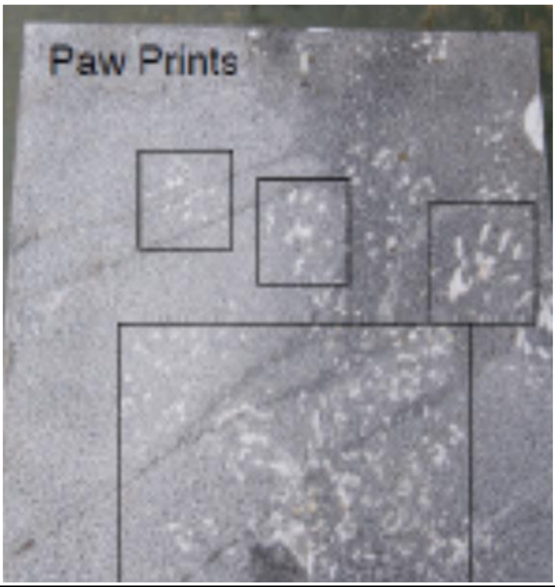

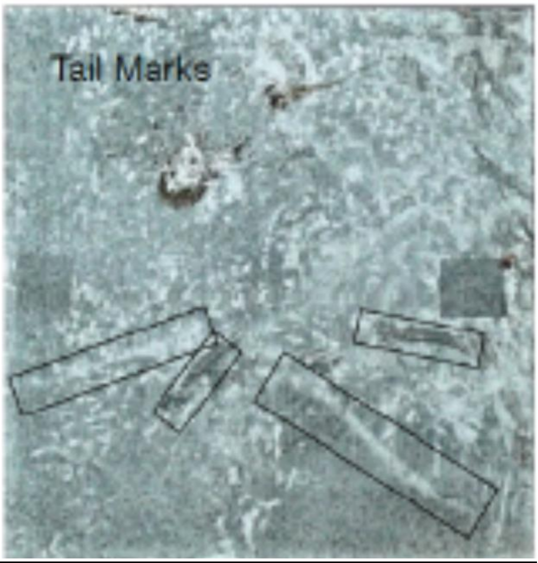
Tail Marks

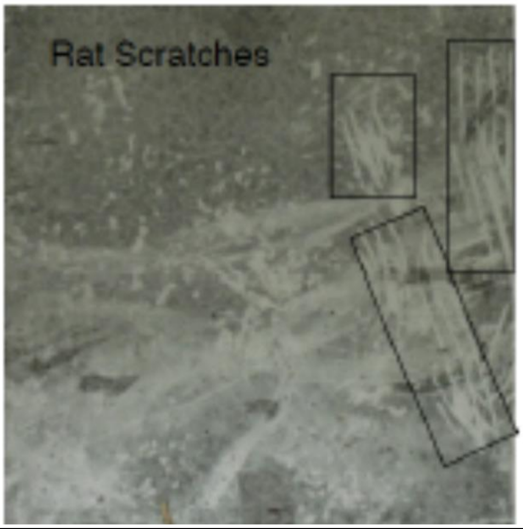
Rat Scratches

Part II - Snap Trapping

Principal objective of snap trapping:

The main objective of rodent trapping is to capture rodent either for the purpose of reducing their population (snap trapping), capture rodents to obtain their organ or tissues for virus/bacteria/parasite isolation/examination or for determining the rodent species richness of a particular location.

Setting and Checking Traps

Materials Needed

- Bait
- Hand gloves
- Victor mouse traps
- Plastic bags
- Backpack

Procedure

Part A - Preparing for trapping expedition

1. If necessary, process the necessary trapping permits/ethical approval for trapping rodent. Obtain information on any endangered species in the anticipated trapping area and learn how to avoid them (preferably avoid conducting trapping in such area if possible).
2. Check the functionality and integrity of the mouse traps.
3. Prepare the bait (probably hotdog or any other good bait) a night prior to the day of trapping.
4. Pack all the required materials a day before the trapping day following the list of required materials.

Park B - Trap placement

1. Arrive at the trapping site in time to set out and bait all traps.

2. Place traps in areas that are relatively out of sight of road users or other areas of high human activity.

3. Do not place traps in areas with high livestock activities to prevent killing of non-targeted species, destruction or accidental tripping of traps.

4. Mark all trapping point on the map

5. Determine the number of mouse traps that should be activated within 5-10m of each location

6. Where possible, place traps near areas with active rodent signs near brush piles, fallen logs, abandoned cars or other items that could provide shelter. When in or near buildings, place traps parallel to and against walls or other vertical surfaces

7. A sketch of the trapping site and trap line placement could be helpful if it is necessary to know the exact site of capture for individual rodents. The location of the trapping site may be recorded on a local topographic map, using GPS if possible.

Part C - Collecting captured rodents

1. Traps should be checked as early in the morning as possible, especially in hot weather and when traps are exposed to direct sun.

2. Team members should wear protective clothing, including long pants and long-sleeved shirt, socks and safety boats.

3. For efficiency and to reduce trap losses, each team member should check the traps he/she set out.

4. Check each trap for evidence of capture or visitation. If a trap appears to have been visited but not sprung (e.g., contains urine, feces, or nesting material in or on the trap), place the trap in a double plastic bag to be decontaminated and checked for proper function. Replace the trap with a clean trap.

5. If the trap contains a target species, note the location of capture and record it as appropriate. Carefully place the trap into a plastic bag and tie. Then place the bagged trap into a second plastic bag and tie it tightly.

6. Transport all captured animals to laboratory for further examination (where necessary) or discards carcasses accordingly following procedure for discarding biological waste.
