# Supplementary material for: Systematic surveillance tools to reduce rodent pests in disadvantaged urban areas can empower communities and improve public health
Source: Sci Rep. 2024 Feb 24;14:4503. doi: 10.1038/s41598-024-55203-5 (PMC10894258; doi:10.1038/s41598-024-55203-5)
Supplement: Supplementary file 6 — Supplementary Information 6. [file 41598_2024_55203_MOESM6_ESM.pdf]

Systematic surveillance tools to reduce rodent pests in disadvantaged urban areas can empower communities and improve public health

Adedayo Michael Awoniyi[1,2†*], Ana Maria Barreto[2†], Hernan Dario Argibay[1], Juliet Oliveira Santana[3], Fabiana Almerinda G. Palma[1], Ana Riviere-Cinnamond[4], Gauthier Dobigny[5,6], Eric Bertherat[7], Luther Ferguson[8], Steven Belmain[9] & Federico Costa[1,2,3,10,11*]

[1]Instituto de Saúde Coletiva, Universidade Federal da Bahia, Salvador - BA, 40110-040, Brasil

[2]Instituto de Biologia, Universidade Federal da Bahia, Salvador - BA, 40170-115, Brasil

[3]Centro de Pesquisas Gonçalo Moniz, Fundação Oswaldo Cruz, Salvador Bahia, Brasil

[4]Data Management, Analytics and Products (DMAP), Health Information and Risk Assessment Unit (HIM), PAHO Health Emergencies, Washington DC USA

[5]French Institute of Research for Sustainable Development (IRD), UMR CBGP, Montpellier, France

[6] Pasteur Institute of Madagascar, Plague Unit, Antananarivo, Madagascar

[7]Department of Pandemic and Epidemic Diseases, World Health Organization WHO, Geneva, Switzerland

[8]Department of Environmental Health Services (DEHS), Ministry of Environment and Natural Resources, Government of The Bahamas

[9]Natural Resources Institute, University of Greenwich, Chatham Maritime, Kent ME4 4TB, UK

[10]Department of Epidemiology of Microbial Diseases, Yale School of Public Health, New Haven, CT06511, USA

[11]Lancaster Medical School, Lancaster University, Lancaster, LA1 4YW, UK

[†]These authors contributed equally and should be considered as co-first authors

***Correspondence to:** AMA | E-mail: maawoniyi13@gmail.com; FC | E-mail: federico.costa@ufba.br

ANNEX VI: Guidelines for the Community Meeting and Collaborative Mapping

Recommendations

- Participation of the participants/community members in the community meeting and collaborative mapping exercise requires that people with different levels of education, gender, socioeconomic levels are involved in the exercise.
- It is recommended that representatives from the same blocks work together as a group (a group can comprise of between 3-5 persons)
- Invite an additional number of people planned for the activity, considering that some of them will miss the community meeting for personal reasons.

Greetings to the Resident

- Say hello, introduce yourself/team (i.e. inform the residents that you are a member of the Bahamas rodent control program)
- Present yourself and the team members (neatly dressed, if possible in a T-shirt with the inscription of the program/project) visibly placed

Check the Residents' availability to listen to the team

- Ask if the resident is available and willing to spare some of his/her time
- Appreciate their collaboration with the team
- Ask if the resident is interested in participating in the community meeting/collaborative mapping
- If they are available, avail them details of the exercise i.e. the place, time, duration and date of the meeting/collaborative mapping
- Answer resident's questions should there is any- clarify the objectives of the study
- Appreciate their collaboration with the team

PART I- Community Meeting

Preparation

❖ Remember the number of people who were invited from the blocks where the team has carried out other rodent control activities, it is recommended to invite at least 8 participants and at most 15 participants

❖ It is advised that the team arrive at the meeting place at least 1 hour before the time scheduled with the participants to:

✔ organize seating arrangement for people to sit in a circle

✔ organize the activity materials

❖ The team must check the material needed to hold the meeting

Start the meeting with a dynamic presentation to the participants

✔ The team (moderator – a member of the research team, and assistant – advisable to use somebody from the community to aid free flow of information) should make a brief presentation to the participating members, informing them of their names, institutional affiliations, objective of the activity and whatever else is necessary (self introduction).

✔ Request that the participants also introduce themselves to the team and to the group informing them of their name, length of time that they have lived in the community, etc. At this point, it is important that a team member writes down all the information regarding the characteristics of the group members, including the minutes of the meeting.

PART II – Community collaboration question scripts

Start the dialogue, with the questions available in the script, allowing the participants to talk about other demands not foreseen in the prepared question script, which should only serve as a guide during the conversation.

Theme: Living in the community

- What is it like living in this community?
- What positive experiences have you had in your community?
- What are the main problems in your community?
    - ➢ Which of the followings do you consider as factor influencing rodent proliferation in your community (unemployment or poor household income, proximity to abandoned property, access to dumpsters, extreme weather events, irregular garbage pick-up schedule by the government, social custom/taboos about rodents, human and animal food availability, others (mention them)
    - ➢ Which of the above factors do you think is mostly related to rodent proliferation in your community?
- Based on the mentioned rodent-associated problems, suggest possible solution(s) that you think is most appropriate to reduce to prevent rodent proliferation in your community
    - ➢ Which of the mentioned solution do you think is the most important to curb rodent problem in your community?

Theme: Perception and exposure to risk

- Do you think rodents are a problem in your community?
- What are the main fears/concerns you have about rodents?
- In your opinion, what contributes to the proliferation of rodents in your community?

- ❖ When preparing the action plan (sample available below), it is suggested that when filling out the document begin with the simplest activities to be carried out that meet the wishes of most community participants.
- ❖ It is suggested that the evaluation of the community meeting be carried out by the participants (with the guidance of the research team where necessary).
- ❖ Following the evaluation, you could ask the following questions
  - What are your opinions about the meeting?
  - What do you evaluate as positive from the meeting's outcome?
  - Do you have suggestions for further meetings?
- ❖ Time for snack
- ❖ Preparation of a report on the meeting for institutional registration.
- ❖ Ensure to deliver a copy to of the report to the community members

Below template could be used to draft the key outcome of the meeting, this should be summarized later by the research team members

ATTACHMENT: ACTION PLAN MODEL

Participant	Problem	Possible solution	Responsible	How[1]	Where[2]	Observation[3]

[1]**How:** the strategies to be used, material and resources, and other sectors required to carry out the planned activity

[2]**Where:** the area where suggested solution is most appropriate

[2]**Observations:** it is suggested to enter information about the progress and updates of activities, etc

PART III - Collaborative mapping questions

- ❖ Greetings: introduce the team to the participants and have them introduce themselves
- ❖ Ask participants to indicate the following on the map using the specified letters
  - Places with the presence of many rodents in your community. Use the letter "**R**" to mark the locations on the printed map

- Places with the presence of a lot of accumulated garbage in your community. Use the letter “**G**” to mark the locations on the printed map – indicate in front of **G** the type of the accumulated garbage e.g. solid waste like derelict vehicle, construction materials, organic garbage etc
- Places that have a lot of abandoned land or property in your community. Use the letter “**ALP**” to mark the locations on the printed map
- Places that have a lot of accumulated rubble in your community. Use the letter “**AR**” to mark the locations on the printed map
- Places that have a lot of vegetation without care in your community. Use the letter “**V**” to mark the locations on the printed map.
- Places with notable decent areas in your community. Use the letter “DA” to mark the locations on the printed map etc

❖ **Theme: Actions to reduce risk**

- What actions have been taken by community residents to control rodents?
- In your opinion, what actions has the government taken to control rodents in your community?
- In your opinion, what could be done by residents to improve rodent control and the problems of accumulated garbage, abandoned land, accumulated rubble and the presence of a lot of neglected vegetation in your community?

Note: Mention the most frequent problems in collaborative mapping.

- What can we do by working together (community and government) to solve the rodent and other problems you have identified in your community?

Note: Mention the most frequent problems in collaborative mapping
